# Impaired Acetyl-CoA Compartmentalization Drives a Futile Lipogenic–Oxidative Cycle in N88S Seipinopathy

**DOI:** 10.3390/cells15050395

**Published:** 2026-02-24

**Authors:** Vítor Moreira, Carlo W. T. van Roermund, Vítor Costa, Vitor Teixeira

**Affiliations:** 1 i3S Instituto de Investigação e Inovação em Saúde, Universidade do Porto, 4200-135 Porto, Portugalvcosta@i3s.up.pt (V.C.); 2 IBMC—Instituto de Biologia Molecular e Celular, Universidade do Porto, 4200-135 Porto, Portugal; 3 Laboratory Genetic Metabolic Diseases, Department of Clinical Chemistry, Amsterdam UMC, University of Amsterdam, Meibergdreef 9, 1105 AZ Amsterdam, The Netherlands; c.vanroermund@amsterdamumc.nl; 4 ICBAS—Instituto de Ciências Biomédicas Abel Salazar, Universidade do Porto, 4050-313 Porto, Portugal

**Keywords:** lipid droplet, seipin, misfolding, seipinopathy, peroxisomes, glyoxylate cycle, mitochondria, acetyl-CoA

## Abstract

**Highlights:**

**What are the main findings?**

- The N88S seipin mutation disrupts acetyl-CoA compartmentalization, limiting its proper utilization through mitochondrial and glyoxylate cycle consuming pathways.
- Misrouting of acetyl-CoA promotes cytosolic lipogenesis and potentiates oxidative stress.

**What are the implications of the main findings?**

- Defective peroxisome–mitochondria metabolic coupling fuels a futile lipogenic–oxidative cycle that amplifies cellular dysfunction in N88S seipinopathy.
- Intervening at multiple metabolic control points, by enhancing mitochondrial acetyl-CoA utilization, promoting phospholipid biosynthetic flux, modulating lipogenic transcriptional programs, and optimizing peroxisomal metabolic capacity, offers novel therapeutic avenues to treat seipinopathies and related motor neuron diseases.

**Abstract:**

The N88S mutation in human seipin causes a dominant motor neuron disease marked by ER stress and inclusion body formation, lipid imbalance, and oxidative damage. However, the metabolic mechanisms connecting these defects remain poorly understood. Previous proteomic profiling in our yeast model of N88S human seipinopathy revealed decreased protein levels of enzymes involved in the tricarboxylic acid cycle, fatty acid and carboxylic acid metabolism, and the glyoxylate cycle, suggesting impaired downstream utilization of peroxisome-derived acetyl-CoA. Guided by these findings, we investigated how peroxisomal function contributes to cellular dyshomeostasis. N88S seipin-expressing cells exhibited increased peroxisome abundance but defective routing of acetyl-CoA into mitochondrial and glyoxylate pathways, resulting in elevated reactive oxygen species (ROS), impaired glyoxylate cycle activation, and reduced metabolic adaptability to non-fermentable carbon sources. Loss of peroxisomes or forced cytosolic redirection of acetyl-CoA further exacerbated ER stress, ROS accumulation, lipid peroxidation, and the growth defect on N88S seipin-expressing cells, whereas inhibition of fatty acid synthesis mitigated oxidative damage. These findings demonstrate that N88S seipin triggers a futile cycle in which misrouted cytosolic acetyl-CoA drives lipogenesis, amplifying oxidative damage and ER stress. We conclude that defective peroxisome–mitochondria metabolic coupling and acetyl-CoA misrouting may represent central pathogenic mechanisms driving cellular dysfunction in N88S-linked seipinopathy.

## 1. Introduction

Lipid droplets (LDs) are conserved organelles responsible for storing neutral lipids, composed of a triacylglycerol (TG) and sterol ester (SE) core surrounded by a phospholipid monolayer enriched with regulatory proteins [1,2,3]. Their correct biogenesis and turnover are critical for maintaining cellular lipid balance, and disturbances in LD dynamics are linked to metabolic and neurodegenerative diseases. A central factor in LD formation is seipin, an ER membrane protein with two transmembrane domains, cytosolic N- and C-terminal ends, and a conserved ER luminal loop, which oligomerizes into a ring-shaped complex essential for TG nucleation and LD budding [4,5,6]. Loss of seipin in yeast or higher eukaryotes results in clustered or enlarged LDs and altered lipid composition [4,5,6]. Conversely, gain-of-function mutations in human seipin, including N88S and S90L, cause autosomal dominant motor neuron diseases (MNDs) such as Silver syndrome, Charcot–Marie–Tooth type 2, and distal hereditary motor neuropathy [7,8,9]. Seipin is expressed in neurons of the spinal cord and cortex, consistent with the progressive degeneration of upper and lower motor neurons in seipinopathy, leading to gait impairment, muscle atrophy, and pes cavus [8]. These N88S and S90L mutations abolish *N*-glycosylation, driving ER aggregation into inclusion bodies (IBs), and activation of the unfolded protein response (UPR), ultimately leading to cell stress and death [10,11,12,13]. Although mutant seipin undergoes ubiquitination and ER-associated degradation (ERAD), persistent ER stress and lipid imbalance suggest additional pathogenic mechanisms [10,11,12,14,15,16]. Using *Saccharomyces cerevisiae* as a model system, our recent work provided the first evidence that oxidative stress constitutes a central pathogenic mechanism in N88S-associated seipinopathy [15,17]. We demonstrated that expression of the N88S mutant protein triggers persistent activation of the redox-sensitive transcription factor Yap1p, accompanied by elevated reactive oxygen species (ROS) levels [16]. We also uncovered key alterations of phospholipid metabolism and iron regulation in the disease process. N88S seipin expression causes phosphatidic acid (PA) accumulation and impaired flux through the CDP-diacylglycerol pathway, resulting in defective phospholipid biosynthesis and misregulation of the transcriptional repressor Opi1p activity, with derepression of *INO1* and increased inositol-3-phosphate synthase levels [15]. Although steady-state levels of major phospholipids remain stable, disruption of lipid homeostasis emerges as a potential contributor to neurotoxicity observed in human seipinopathy, where misfolding of the N88S seipin variant serves as the initiating event [15]. This is consistent with the established role of seipin as a central regulator of both phospholipid balance and neutral lipid metabolism. In addition, lysophospholipids accumulate [15], and their signaling has been implicated in neuronal dysfunction, consistent with their established involvement in Alzheimer’s disease, Parkinson’s disease, and demyelinating disorders [18,19,20]. These findings suggest that PA and lysophospholipids could serve as potential biomarkers or therapeutic targets for seipinopathy. Iron homeostasis is also perturbed in N88S seipin-expressing cells, as Aft1p/Aft2p-dependent transcription of iron uptake and storage genes is impaired, leading to reduced levels of Fit1p, Arn1p, Arn2p, and Hmx1p, alongside abnormal regulation of Fet3p [15]. This imbalance correlates with reduced aconitase activity, indicating defects in mitochondrial Fe-S cluster assembly and susceptibility to ROS [15]. These results support the concept that seipinopathy represents both a proteinopathy and a lipidopathy, where defective lipid metabolism and iron imbalance act synergistically to promote ER stress and oxidative damage.

Peroxisomes are dynamic organelles required for various metabolic processes, and their biogenesis is orchestrated by a conserved set of peroxins [21]. Among these, Pex3p and the cytosolic chaperone–receptor Pex19p play essential roles in the early steps of membrane formation, targeting, and insertion of peroxisomal membrane proteins, thereby ensuring the establishment of functional organelles [21,22]. In yeast, peroxisomes are central hubs of lipid metabolism, harboring the enzymes of the fatty acid (FA) β-oxidation pathway, including acyl-CoA oxidase Pox1p and 3-ketoacyl-CoA thiolase Pot1p, which catalyze the sequential degradation of FAs. This process generates acetyl-CoA as a key metabolic intermediate, linking peroxisomal activity to broader cellular networks of carbon utilization [23]. The acetyl-CoA produced in peroxisomes can enter the tricarboxylic acid (TCA) cycle in mitochondria to sustain energy production, or it can be directed into the glyoxylate cycle to conserve carbon skeletons during growth on non-fermentable carbon sources [24]. The glyoxylate cycle, absent in mammals but essential in yeast, functions as an anaplerotic route that replenishes TCA intermediates while conserving carbon skeletons for biosynthesis. This pathway relies on the enzymes isocitrate lyase (Icl1p) and malate synthase (Mls1p), which redirect acetyl-CoA into four-carbon dicarboxylic acids that can feed gluconeogenesis and anabolic metabolism [25,26]. A central player in this pathway is citrate synthase 2 (Cit2p), a peroxisomal isoform of citrate synthase, which condenses acetyl-CoA with oxaloacetate to produce citrate, providing a crucial entry point that connects peroxisomal metabolism to both mitochondrial and cytosolic networks [27,28]. Through this mechanism, the glyoxylate shunt allows yeast cells to grow on FAs, acetate, or ethanol, ensuring metabolic flexibility under nutrient-limiting conditions. Mitochondria act as integrative platforms that receive acetyl-CoA from glycolysis, peroxisomal β-oxidation, and other sources to fuel the TCA cycle, oxidative phosphorylation, and biosynthesis. In addition to ATP generation, mitochondria are critical for Fe-S cluster assembly, redox regulation, and ROS detoxification, functions that are essential for cellular survival [29,30]. Together, peroxisomes, mitochondria, and the ER form interconnected metabolic systems that coordinate acetyl-CoA partitioning, balance catabolic and anabolic fluxes, and enable yeast cells to adapt to diverse nutrient conditions.

Building on our previous multi-omics analysis using mass spectrometry in this yeast model of human N88S seipinopathy, we hypothesized that peroxisomal dysregulation contributes directly to cellular dysfunction and the pathogenesis of N88S seipinopathy [15]. Collectively, our findings indicate that N88S seipin-induced misrouting of acetyl-CoA triggers a futile cycle of aberrant lipogenesis and oxidative stress, establishing disrupted acetyl-CoA compartmentalization as a central mechanistic driver of cellular pathology of the disease.

## 2. Materials and Methods

### 2.1. Yeast Strains and Plasmids

The *S. cerevisiae* strains utilized in this study were derived from the W303α parental strain and are detailed in Table S1. Using standard PCR-based homologous recombination techniques, processes like protein tagging and gene deletions were carried out [31,32]. Primers for these procedures were designed using the Primers-4-Yeast tool [33] in conjunction with pFA6 plasmid set [31]. Plasmids used in this study are listed in Table S2. For cloning of ADH1pr-MPC1-ADH1t into the pRS315-UPRE-LacZ vector [16], the plasmid UG75-ADH-CDS1-3HA [34] was digested with ClaI and NheI and MPC1 ORF was amplified by PCR, using BY4741 genomic DNA as template, and integrated into the same sites of the plasmid using specific primers. Next, this newly generated plasmid was digested with SacII, and the resulting insert was cloned into the SacII restriction site of the pRS315-UPRE-LacZ vector. For cloning of ADH1pr-CAT2_cyt_-ADH1t into the pRS315-UPRE-LacZ vector [16], the plasmid UG75-ADH-CDS1-3HA was digested with BlpI and NheI and CAT2 gene variants were amplified by PCR, using BY4741 genomic DNA as template, and integrated into the same sites of the plasmid using specific primers. Next, this newly generated plasmid was digested with SacII, and the resulting insert was cloned into the SacII restriction site of the pRS315-UPRE-LacZ vector. CAT2 encodes a protein with a mitochondrial targeting signal (MTS) at the N-terminus and a peroxisomal targeting signal (PTS) at the C-terminus (the tripeptide AKL). Here, the full-length CAT2 ORF encodes a mitochondrial precursor protein of 670 amino acids, which is designated as CAT2_mit_. We also constructed pRS315-UPRE-LacZ-ADH1pr-CAT2_cyt_-ADHt, in which the PTS and the nucleotides encoding the first 22 amino acids of CAT2 were deleted. The protein encoded by this construct is located both in the peroxisomes as well as the cytosol, and it was named cytosolic CAT2 (CAT2_cyt_). To monitor CIT2-LacZ expression, the indicated strains contained a EagI-cut CIT2-lacZ reporter gene integrated at the CIT2 locus. All constructs were verified either by sequencing (plasmids) or PCR (mutant strains). Strains were transformed using the standard lithium acetate procedure [35].

### 2.2. Culture Media and Growth Conditions

Yeast cells were grown under aerobic conditions at 26 °C in Erlenmeyer flasks placed on a gyratory shaker set to 140 rpm. The growth medium volume was maintained at a 1:5 ratio relative to the flask volume. The liquid growth media used for yeast cultivation consisted Yeast Peptone Dextrose (YPD) containing 1% (wt/vol) yeast extract (Conda Pronadisa, Madrid, Spain), 2% (wt/vol) bacto peptone (LabM), and 2% (wt/vol) glucose (Fisher Scientific, Hampton, NH, USA), Synthetic Complete (SC) Medium composed of 2% (wt/vol) of the indicated carbon source like glucose (Fisher Scientific), raffinose (Biosynth Ldt, Newbury, United Kingdom) or sodium acetate (Thermo Scientific, Waltham, MA, USA) and 0.67% (wt/vol) yeast nitrogen base (YNB) without amino acids (BD BioSciences, Franklin Lakes, NJ, USA), supplemented with the following amino acids and nucleotides: 0.008% (wt/vol) histidine (Sigma Aldrich, St. Louis, MO, USA), 0.008% (wt/vol) tryptophan (Sigma Aldrich), 0.04% (wt/vol) leucine (Sigma Aldrich), 0.008% (wt/vol) uracil (Sigma Aldrich), and 0.008% (wt/vol) adenine (Sigma Aldrich). For solid versions of previous media, 1.5% (wt/vol) agar (Conda Pronadisa) was added. Where indicated cerulenin (C2389, Merck KGaA, Darmstadt, Germany) was added to a final concentration of 1 µg/mL for the inhibition of fatty acid synthase (FAS).

### 2.3. Bioinformatics Analysis

Gene ontology (GO) and transcriptional regulatory analyses of differentially expressed proteins (DEPs) were performed using the YEASTRACT+ platform (https://www.yeastract.com/, accessed on 20 March 2024). DEPs were mapped to their corresponding Saccharomyces cerevisiae genes and analyzed using the YEASTRACT+ functional enrichment tools, which are based on manually curated transcriptional regulatory associations. GO enrichment analysis was conducted to identify significantly overrepresented biological process categories among the DEPs compared to the whole yeast genome. Statistical significance was evaluated using the default enrichment algorithms implemented in YEASTRACT+, and GO terms with adjusted *p*-values were considered significant (*p* ≤ 0.05)

### 2.4. β-Galactosidase Activity Assay

Cells containing LacZ-reporter fusion plasmids were cultured in SC medium. After growth, the cells were collected by centrifugation, resuspended in breaking buffer (100 mM Tris, 1 mM DTT, 10% (vol/vol) glycerol) supplemented with protease inhibitors (Complete Mini EDTA-free Protease Inhibitor Cocktail tablets, Sigma Aldrich), and mechanically disrupted using zirconium beads for 5 min. The cell debris was removed by centrifugation at 14,000× *g* for 15 min at 4 °C, and the supernatant was collected for protein quantification. Total protein concentrations were determined using the Lowry method, with a standard curve generated from bovine serum albumin. Aliquots containing 15–100 µg of total protein were diluted to a final volume of 800 µL with β-galactosidase assay buffer (60 mM Na_2_HPO_4_, 40 mM NaH_2_PO_4_, 10 mM KCl, 1 mM MgSO_4_, 50 mM β-mercaptoethanol). The samples were then incubated at 30 °C for 5 min in the presence of 200 µL of the substrate o-nitrophenyl-β-D-galactopyranoside (ONPG; Merck, Kenilworth, NJ, USA), as previously described [36].

### 2.5. Western Blotting Analysis

To evaluate the total protein levels of Pex5p, Pot1p, acetyl-H3 and histone-H3 in WT-VN WT-VC and N88S-VN N88S-VC strains, cells were cultured until they reached either the exponential (EXP) or post-diauxic shift (PDS) phase in SC-glucose medium at 26 °C. The cells were then washed and collected by centrifugation at 4000 rpm for 4 min at 4 °C. Protein extraction was performed using alkaline lysis, and the samples were prepared in Laemmli sample buffer. Proteins were separated by SDS-PAGE using 10% polyacrylamide gels for detecting Pex5p, Por1p, histone-H3 and acetyl-H3 total levels. The separated proteins were transferred to nitrocellulose membranes (Hybond-ECL, GE Healthcare, Chicago, IL, USA) using a semi-dry transfer system for 1 h. For immunodetection of Pex5p, Pot1p and histone-H3, the membranes were blocked with 5% (wt/vol) nonfat dry milk in TTBS buffer (20 mM Tris, 140 mM NaCl, 0.05% (vol/vol) Tween-20, pH 7.6) for 1 h at room temperature. For the immunodetection of acetyl-H3, membranes were blocked with 5% (wt/vol) BSA in the TTBS buffer. The membranes were then incubated with primary antibodies—polyclonal rabbit anti-Pex5p, anti-Pot1p antibody (1:10,000 dilution, Prof. Ralf Erdmann), anti-histone-H3 and anti-acetyl-H3 (1:3000 dilution, abcam-ab1791 and Cell Signalling mAB #7627, respectively)—overnight at 4 °C. After washing with TTBS, the membranes were incubated with an anti-rabbit secondary antibody (1:5000 dilution), 1 h at room temperature. Immunodetection was carried out by chemiluminescence using WesternBright ECL reagent (Advansta Inc., San Jose, CA, USA), and LucentBlue X-ray films (Advansta). Band intensities were quantified using a GS-900 Calibrated Densitometer (Bio-Rad, Hercules, CA, USA). For membrane stripping, the membranes were washed with TTBS and incubated in stripping buffer (62.5 mM Tris-HCl, pH 6.8, 2% (wt/vol) SDS, 10 mM 2-mercaptoethanol) for 30 min at 50 °C. the blots were visualized using enhanced chemiluminescence reagents (Perkin Elmer Inc., Waltham, MA, USA), and band intensities were analyzed using ImageJ software v2.0.0-rc-69/1.52p (National Institute of Health, Bethesda, MD, USA).

### 2.6. Fluorescence Microscopy

To examine the intracellular localization of IBs using the Venus signal upon reconstitution of the VN and VC fragments and peroxisomes (using *PTS1*-mCherry), cells were grown to the exponential phase in SC-glucose medium. The cells were centrifuged at 13,400 rpm during 1 min, then washed with 1 mL of water, centrifuged again in same conditions and resuspended in 25 µL of SC-glucose and finally visualized by fluorescence microscopy (Zeiss Axio Imager Z1 Apotome, Carl Zeiss, Oberkochen, Germany or Leica TCS SP8, Leica Microsystems, Wetzlar, Germany). Z-stacks were captured for the DIC, mCherry, and Venus channels. Final images and quantification analyses were processed using ImageJ software (version 2.0.0-rc-69/1.52p). Where applicable, all quantifications were based on at least two independent experiments, with over 100 cells evaluated per condition. Data were recorded in Excel (Microsoft v.16.105.1, Redmond, WA, USA) and analyzed using Prism 8.0 (GraphPad Software v11.0.0, Boston, MA, USA). Adjustments to brightness and contrast were made using Inkscape (The Inkscape Project v1.4.2., free source).

### 2.7. ROS Staining

To assess ROS, cells grown in SC-glucose medium at specified phases were incubated with 5 μg/mL dihydroethidium (DHE, Molecular Probes, Eugene, OR, USA) for 10 min at room temperature in the dark. Then, cells were centrifuged, washed twice and resuspended in PBS [16]. Flow cytometry analysis was performed using the FL3 (670 LP) channel (BD Accuri C6 Flow cytometer, BD Biosciences) for ROS quantification. Data were evaluated with FlowJo software (v. 10.6.1, Ashland, OR, USA).

### 2.8. Yeast Spotting Assay

Growth assays were conducted by applying serial 1:10 dilutions of exponentially-grown cell cultures onto SC-glucose or SC-acetate plates with or without sodium arsenite (NaAs) at the final concentration of 250 μM. Plates were incubated for 2 days or 5 days at 26 °C.

### 2.9. β-Oxidation Measurement

β-oxidation activity was measured using 11 µM of [1-^14^C]-labelled octanoate (C8:0) as substrate (American Radiolabeled Chemical, St. Louis, MO, USA), as described previously [37]. The sum of the end products of β-oxidation, which includes [^14^C]-labelled CO_2_ and acid-soluble products (ASP), was taken as a measure of β-oxidation activity.

### 2.10. Enzymatic Activity Assays

Cells were grown to PDS phase in SC-glucose medium and harvested by centrifugation for 5 min at 4000 rpm (4 °C). Cells were resuspended in their respectively buffer, as indicated in Table S3, containing protease inhibitors (Complete, EDTA-free Protease Inhibitor Cocktail, Roche, Basel, Switzerland). The protein extracts were obtained by mechanical disruption of the cells by vortexing in the presence of glass beads for 10 min. Cell debris was removed by centrifugation at 3000 rpm for 15 min at 4 °C, and protein concentration was determined by the method of Lowry, using bovine serum albumin (BSA) as a standard. Measurements of the activities of enzyme were done spectrophotometrically at 25 °C adding the respective reagents (Table S3) as described in [38]. One unit of enzyme activity was defined as 1 nmol of protein sample converted per minute and per mg of protein. Acetyl–coenzyme A synthetase activity was determined as previously described [39]. Cells were grown to PDS phase in SC-medium lacking histidine and leucine, and 0.2 mg protein extract or the equivalent volume of buffer was used (control).

### 2.11. Lipid Peroxidation Assay

For lipid peroxidation analysis, cells were grown in SC-glucose lacking histidine and leucine to stationary phase. Yeast extracts were prepared in 20 mM sodium phosphate buffer (pH 7.2), by vigorous shaking of the cell suspension, in the presence of glass beads, for 5 min. Trichloroacetic acid (10% wt/vol) was added and two more pulses of 1 min were performed. Total protein levels were quantified by the Lowry method using a bovine serum albumin standard curve. Lipid peroxidation was assayed in 600 μL of 1% (wt/vol) thiobarbituric acid, 0.05 M NaOH, 0.025% (wt/vol) butylated hydroxytoluene, 100 μL of 0.1 M EDTA, and 100 μL of total protein extract. Malondialdehyde (MDA) concentration was determined spectrophotometrically at 532 nm and expressed as nanomoles of MDA. (mg of protein)^−1^ [40].

### 2.12. Measurement of Glutathione Levels

Cells were grown in SC-glucose lacking histidine and to PDS phase. The Glutathione GSH/GSSG Assay Kit (MAK 440, Sigma Aldrich) was used to measure total, reduced and oxidized glutathione levels in samples using an enzymatic method that utilizes Ellman's Reagent (DTNB) and glutathione reductase. A total of 10 OD_600_ cells were used to perform the measurements according to the manufacturer’s instructions. Yeast extracts were prepared in 20 mM sodium phosphate buffer (pH 7.2), by vigorous shaking of the cell suspension, in the presence of glass beads, for 5 min.

### 2.13. Statistical Analysis

Unless otherwise stated, the results were obtained from at least three independent experiments. The images displayed are representative of these outcomes. Quantitative data are expressed as the mean ± standard deviation (SD). Statistical analyses were performed using unpaired, two-tailed Student’s *t*-tests or one-way ANOVA, carried out with Prism 8.0 software (GraphPad Software v11.0.0, MA, USA). *p*-values < 0.05 were considered significant: * *p* ≤ 0.05; ** *p* ≤ 0.01; *** *p* ≤ 0.001; **** *p* ≤ 0.0001.

## 3. Results

**Figure 1:**
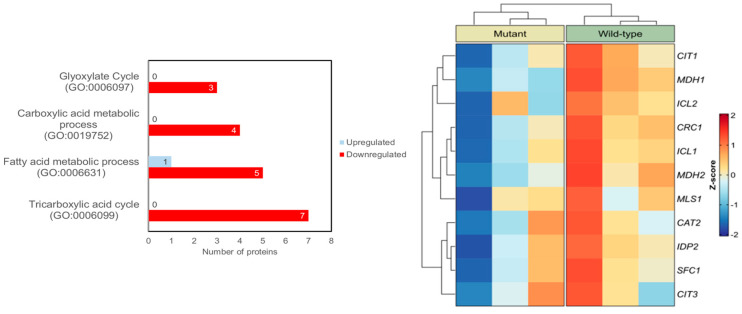
GO-based functional categorization of DEPs with respect to biological processes. Proteins that were significantly altered in N88S seipin-expressing cells were subjected to Gene Ontology (GO) enrichment analysis within the indicated biological process categories (left panel). Heat map analyses of total protein level changes of enzymes and metabolite transporters pathway in WT and N88S seipin-expressing cells (right panel, for 3 independent experiments per strain). Data from [15].

Here, we used a well-established yeast model of human N88S-linked seipinopathy previously developed in our laboratory via a bimolecular fluorescence complementation (BiFC) strategy [16]. This yeast model was constructed in a seipin-deficient background (*sei1*Δ *ldb16*Δ), in which human wild-type (WT) or N88S mutant forms of seipin were expressed as fusions to either the N-terminal (VN) or C-terminal (VC) fragments of the Venus fluorescent protein [16]. Quantitative untargeted mass spectrometric proteomic analysis was previously conducted to examine changes in protein abundance in wild-type (WT) versus N88S seipin-expressing mutant cells [15]. Using YEASTRACT+, a gene ontology (GO) analysis for differentially expressed proteins (DEPs) revealed an enrichment in proteins related to tricarboxylic acid cycle (TCA) (GO:0006099; *p*-value = 5.34 × 10^−8^), fatty acid (FA) metabolic process (GO:0006631; *p*-value = 1.29 × 10^−5^), carboxylic acid metabolic process (GO:0019752; *p*-value = 2.17 × 10^−6^) and glyoxylate cycle (GO:0006097; *p*-value = 2.49 × 10^−6^) (Figure 1). Peroxisomes support cellular metabolism by linking FA and carboxylic acid metabolism with the glyoxylate and TCA cycles to provide metabolic intermediates and support energy homeostasis. Altogether, we posit that peroxisome function is altered in N88S seipin-expressing cells, possibly contributing to alterations in lipid metabolism and flux observed in the mutant [15].

### 3.1. Absence of Peroxisomes Lead to an Increase in IB Formation

**Figure 2:**
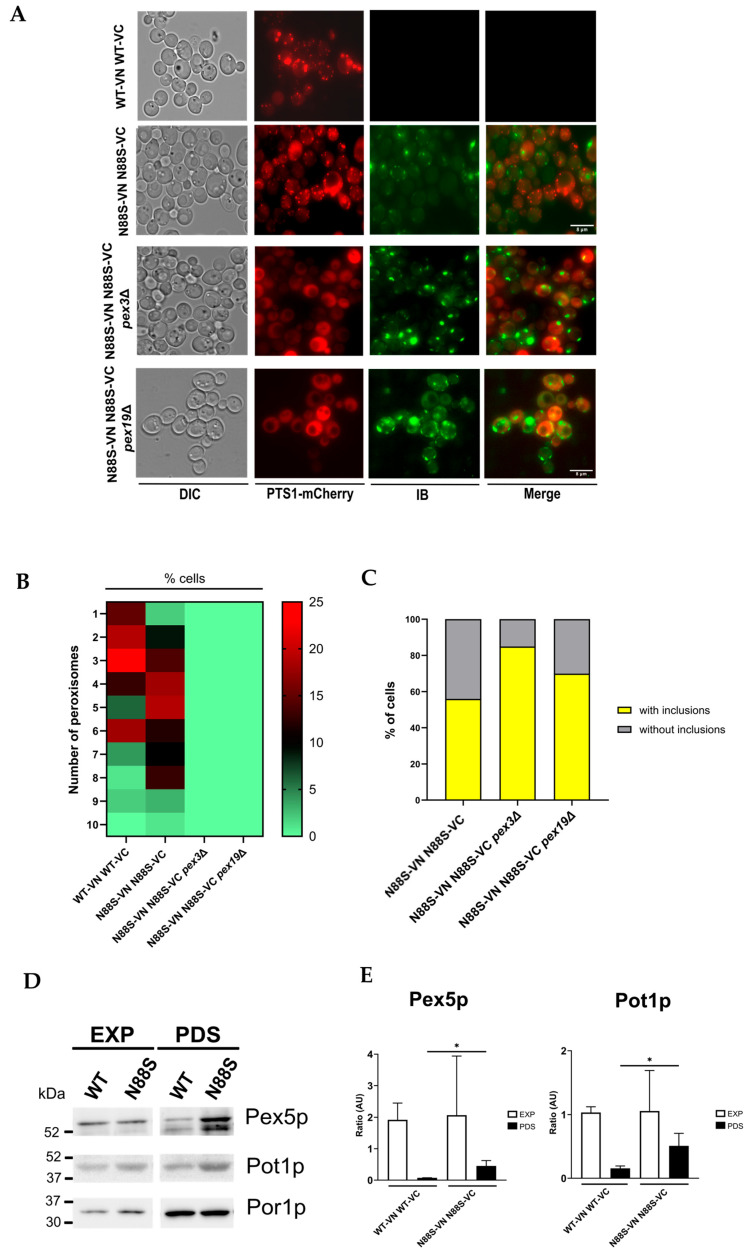
*PEX3* or *PEX19* deficiency increased IBs formation in cells expressing the N88S seipin mutation. (**A**) Formation of IBs was monitored by fluorescence microscopy using the YFP/Venus channel (third column), and peroxisome number and morphology was assessed using a *PTS1*-mCherry fusion construct, in cells grown to the exponential phase in SC-glucose medium. DIC: Differential interference contrast. Bar scale, 8 μm. (**B**) Heat map illustrating the frequency distribution of peroxisome number per cell. The *y*-axis denotes the number of peroxisomes per cell, while the color scale represents the corresponding cell counts. Lower frequencies are depicted in green, and higher frequencies in red. Data were combined from 2 independent experiments. (**C**) Quantification is defined as percentage of cells displaying IB foci (*n* > 100 cells). Data were combined from 2 independent experiments. (**D**) WT and N88S seipin-expressing cells were grown in SC-glucose medium and allowed to reach the exponential (EXP) and post-diauxic shift (PDS) phases. Aliquots were collected at these stages, and Western blot analysis were performed for assessment of Pex5p and Pot1p levels. Por1p was used as loading control (left panel). (**E**) Quantification is based on the ratio between the indicated protein and the loading control as measured by densitometry (right panel). * *p* ≤ 0.05 (unpaired *t*-test).

We focused on investigating the relationship between peroxisome morphology, number, and IB accumulation in WT and N88S seipin-expressing cells. To achieve this, we used fluorescence microscopy, tracking the Venus signal to monitor IB formation as previously described [16], and the PTS1-mCherry signal was used as a peroxisomal marker. The results revealed that N88S seipin mutant cells exhibited a higher number of peroxisomes (Figure 2A,B) than WT cells, although their overall morphology remained similar. To explore the correlation between peroxisomal genes and IB formation, we generated peroxisome *pex3*- or *pex19*-deficient cells. These cells are defective in peroxisomal membrane biogenesis and therefore lack functional peroxisomes, and all matrix enzymes reside in the cytosol [41]. In fact, PTS1-mCherry was observed in the cytosol in N88S mutant cells in both genetic backgrounds (Figure 2A). Importantly, the lack of functional peroxisomes markedly increased IB formation in N88S seipin-expressing cells (Figure 2C), indicating a metabolic link between peroxisomes and IB generation. Together, these findings indicate that expression of N88S seipin increases peroxisome abundance, and that peroxisomal function is required to restrain IB formation.

The observed increase in peroxisome number in cells expressing the N88S seipin mutation (Figure 2A–C) prompted us to examine changes in the levels of Pex5p and Pot1p, which are key proteins involved in peroxisomal biogenesis and function, respectively. Pex5p mediates the import of newly synthesized proteins containing a PTS1 signal into peroxisomes, while Pot1p is a crucial enzyme for FA β-oxidation, reflecting peroxisomal metabolic activity [42]. Western blot analyses revealed higher levels of Pex5p and Pot1p at post-diauxic (PDS) phase (Figure 2D,E) for N88S seipin-expressing cells when compared to its WT counterpart, which corroborate with peroxisome proliferation. This indicates that peroxisomes are fully functional in cells carrying the N88S seipin mutation.

### 3.2. Peroxisome Deficiency Increases Ros Production and Disrupts Redox Balance Without Inducing Er Stress

**Figure 3:**
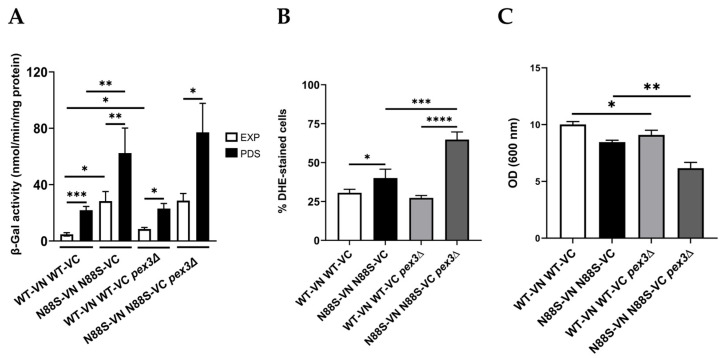
*PEX3* deficiency has no effect in the activation of the UPR in N88S seipin-expressing cells. (**A**) Cells with the specified genotypes expressing UPRE-LacZ were grown in SC-glucose medium lacking leucine and allowed to reach the exponential (EXP) and post-diauxic shift (PDS) phases. At least 3 independent experiments for each strain were performed. Protein extracts were prepared, and specific β-galactosidase (β-Gal) activity was measured using o-nitrophenyl-β-D-galactopyranoside (ONPG) as a substrate, with the amount of o-nitrophenol released indicating enzyme activity. (**B**) Cells with the indicated genotypes were grown in SC-glucose medium until stationary phase, and ROS levels were assessed in cells labeled with dihydroethidium (DHE) via flow cytometry using the FL3 channel. Data were generated from at least 3 independent experiments for each strain. (**C**) Overnight precultures of cells grown in SC-glucose medium were diluted to OD_600_ = 0.15 in fresh medium. Cell density (OD_600_) was measured after a 48 h incubation period. Both graphs were obtained from at least 3 independent experiments for each strain. * *p* ≤ 0.05; ** *p* ≤ 0.01; *** *p* ≤ 0.001; **** *p* ≤ 0.0001 (unpaired *t*-test with Welch’s correction).

Peroxisomes are dynamic, multifunctional organelles that play a central role in maintaining cellular homeostasis by coordinating lipid metabolism with adaptive stress responses. To further understand the potential role of peroxisomes in the disease, we started by examining their impact on key phenotypes, including the ER stress response. We then analyzed how PEX3 deficiency affects the ER stress response, using an UPRE-LacZ reporter. Consistent with earlier observations, cells expressing the N88S seipin variant exhibited increased β-galactosidase (b-gal) activity of the reporter fusion when compared to its WT counterpart at all growth stages (Figure 3A, which correlates with higher ER stress levels [15,16]. Importantly, PEX3 deletion did not significantly alter UPR activation in WT- and N88S seipin-expressing cells (Figure 3A), suggesting that changes in peroxisome number and metabolism are not associated with the induction of the ER stress response in the mutant strain.

Peroxisomes are key organelles that maintain cellular redox balance and homeostasis by linking lipid metabolism with ROS detoxification. We previously demonstrated that ROS levels were elevated in N88S seipin-expressing cells [15,16]. Here, we examined whether alterations in peroxisome dynamics influence ROS production in these cells. For this purpose, cells were grown to the stationary phase, and ROS levels were measured using the dihydroethidium (DHE) probe. Our results showed that deletion of PEX3 increased ROS levels by approximately 75% in N88S cells, while ROS levels in WT cells remained unaffected (Figure 3B). These findings suggest that increased number of peroxisomes may help mitigate intracellular oxidation in cells expressing the N88S seipin mutation. Finally, growth measurement assays comparing WT and N88S cells with their respective pex3-deficient counterparts revealed that loss of peroxisomes impaired growth, with the effect being more pronounced in N88S cells (Figure 3C). Together, these findings indicate that peroxisome dynamics is critical for managing oxidative stress and IB formation, and for sustaining growth in the yeast model of N88S seipinopathy. Peroxisomes contribute to cellular redox homeostasis in part by regulating glutathione levels, a key antioxidant that protects against oxidative stress [43]. Based on this, we evaluated the levels of glutathione. Glutathione exists in two states: oxidized (GSSG) and reduced (GSH) glutathione. The results showed that total glutathione levels were increased in the N88S *pex3*D mutant when compared to N88S seipin-expressing cells (Figure S1). Although no differences were observed in GSH levels and in the ratio GSSG/GSH between WT cells and the N88S seipin mutant, GSSG levels were lower in N88S seipin-expressing cells (Figure S1). The observation of increased ROS concurrent with normal GSH levels and a reduced GSSG pool implies that the glutathione system is actively maintaining redox homeostasis in the mutant, partially compensating for impaired antioxidant defenses, including the previously reported decrease in catalase activity in N88S seipin-expressing cells. Possible explanations for this result include enhanced glutathione reductase activity and/or export of GSSG, which would prevent accumulation of intracellular GSSG. Altogether, these findings indicate that redox imbalance observed in the yeast model of human N88S seipinopathy might be linked to alterations in glutathione metabolism.

### 3.3. N88S Seipin-Expressing Cells Exhibit Increased Peroxisomal Biogenesis Proteins

**Figure 4:**
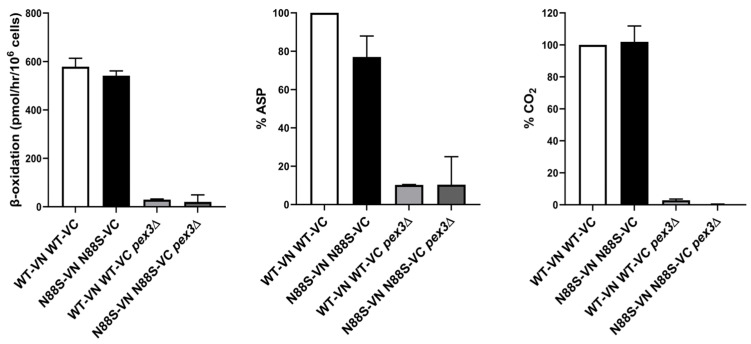
β-oxidation activity is not altered in N88S seipin-expressing cells. FA β-oxidation was assessed using radiolabeled [1-^14^C] octanoate (C8:0) as a substrate and quantified as described in Materials and Methods (left graph). Following extraction, the ASP-containing supernatant (middle graph) and CO_2_-containing supernatant (right figure) were transferred to liquid scintillation vials, and radioactivity was quantified using a liquid scintillation counter (LSC). ASP and CO_2_ production in mutant strains were normalized relative to the WT strain, which was set at 100%. Two independent experiments were performed for each strain.

In order to further analyze peroxisomal function, we determined the activity of the β-oxidation using radiolabeled [1-^14^C]-labelled octanoate (C8:0) as substrate, which was oxidized to acid-soluble products (ASPs) and CO_2_. To achieve this, we used *PEX3*-deficient strains as negative controls and conducted comparative analyses between the WT and N88S strains. The results revealed that although FA β-oxidation rates are similar between WT and N88S mutant cells (Figure 4), ASP levels are decreased by ~30% in N88S seipin-expressing cells while no difference was observed for %CO_2_ generated in TCA cycle (Figure 4). It was previously shown that [1-^14^C]-labelled octanoate (C8:0) is primarily oxidized to oxaloacetate and malate ASPs, which are glyoxylate and TCA cycle metabolites, and carbon dioxide (CO_2_) [44]. This observation indicates that the metabolic flux and incorporation of acetyl-CoA generated from FA β-oxidation into the glyoxylate and TCA cycles are likely impaired (Figure 4).

### 3.4. Downregulation of Glyoxylate Cycle Enzymes Protein Levels Is Not Related to Changes in Their Enzymatic Activity

**Figure 5:**
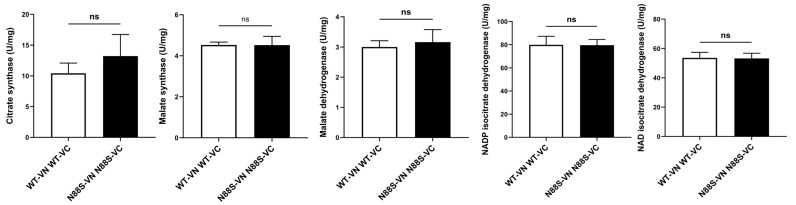
Activities of glyoxylate and TCA cycle enzymes were not affected in N88S mutant strains. Enzyme activities were measured in PDS-grown cells cultured in SC-glucose medium. Data represents the mean ± SD from at least three independent experiments per strain. ns—non-significant (unpaired *t*-test with Welch’s correction).

Previous observations indicated altered ASP generation from FA β-oxidation in N88S seipin-expressing cells. In addition, proteomic profiling [15] and KEGG analysis revealed that the levels of proteins involved in glyoxylate cycle (Icl1p, Mls1p, Mdh2p, Cit2p) and TCA cycle (Cit1/3p, Mdh1p) are significantly reduced (Figure 1). This suggests that although peroxisomal β-oxidation remains active, reduced flux through downstream metabolic pathways may limit the mobilization and conversion of acetyl-CoA into gluconeogenic or energy-yielding intermediates, potentially compromising energy homeostasis and metabolic adaptability under stress conditions. To explore whether decreased protein levels correlate with functional impairment, we measured basal activities of key enzymes from these pathways, including citrate synthase (TCA), malate dehydrogenase (glyoxylate/TCA), malate synthase (glyoxylate), NAD-dependent isocitrate dehydrogenase (mitochondrial), and NADP-dependent isocitrate dehydrogenase (cytosolic) in cells grown to PDS phase in SC-glucose medium, thus matching the conditions used for our previous proteomic analysis [15]. The results revealed no significant changes between WT and N88S seipin-expressing cells (Figure 5). These findings indicate that, despite reduced enzyme abundance, flux through these pathways may remain largely unchanged under basal conditions, likely due to compensatory mechanisms. However, a diminished enzyme pool (Figure 1) may constrain the metabolic flexibility of the cell, thereby limiting its capacity to adjust in response to alterations in carbon source and energy demand.

**Figure 6:**
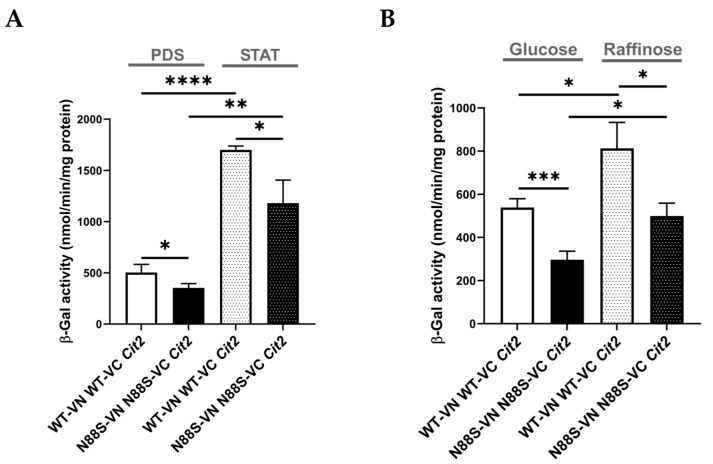
*CIT2*-LacZ expression is reduced in N88S mutant cells under both basal and non-fermentable growth conditions. Cells of the indicated genotypes carrying CIT2-LacZ were grown in SC-glucose medium and harvested at the post-diauxic shift (PDS) or stationary (STAT) phases (**A**), or shifted from SC-glucose to SC-raffinose medium and incubated for 6 h (**B**). Protein extracts were prepared, and specific β-galactosidase (β-Gal) activity was quantified using o-nitrophenyl-β-D-galactopyranoside (ONPG) as a substrate, with o-nitrophenol release reflecting enzyme activity. Data represent mean ± SD from at least three independent experiments per strain. * *p* ≤ 0.05; ** *p* ≤ 0.01; *** *p* ≤ 0.001; **** *p* ≤ 0.0001 (unpaired *t*-test with Welch’s correction).

**Figure 7:**
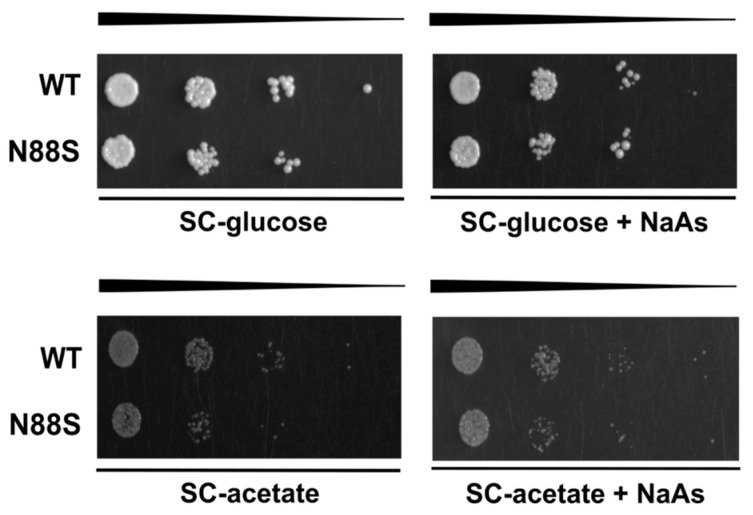
N88S seipin mutant cells display growth comparable to WT in the presence of sodium arsenite (NaAs) independently of the carbon source. Cells with the indicated genotypes were grown in SC-glucose medium to the exponential phase and diluted to OD_600_ = 0.1. Tenfold dilution series were spotted onto SC-glucose or SC-acetate plates supplemented (or not) with sodium arsenite (NaAs, 250 μM). Plates were incubated at 26 °C for 2–5 days.

In yeast, enzymes required for the glyoxylate cycle are upregulated when cells grow on non-fermentable carbon sources (e.g., acetate or raffinose) or during the diauxic shift. Under these conditions, mitochondrial respiration becomes essential for generating energy from acetyl-CoA via the TCA cycle. This adaptive response underscores the physiological relevance of signaling pathways that adjust metabolism in response to mitochondrial function. Numerous nuclear genes are transcriptionally regulated by the mitochondrial state, namely *CIT2*, which encodes a glyoxylate cycle isoform of citrate synthase. Its transcription is strongly induced in response to loss of mitochondrial function through a process known as retrograde (RTG) pathway [45]. Here we investigated how cells adapt to carbon source-dependent stress during PDS and stationary (STAT) phases, periods characterized by metabolic reprogramming, including reversal of carbon flow through glycolysis and activation of the glyoxylate cycle, as well as during a shift to raffinose as the sole carbon source. To assess this, we introduced a *CIT2*-LacZ reporter into WT and N88S seipin-expressing cells and measured β-galactosidase activity. In WT cells, *CIT2*-LacZ expression significantly increased upon transition from PDS to STAT phase, coinciding with glucose exhaustion (Figure 6A). In contrast, the β-gal reporter activity in N88S seipin-expressing cells was reduced by ~30% at both PDS and STAT phases, indicating impaired metabolic adaptation already under basal conditions. This defect became even more pronounced when cells were shifted to SC-raffinose medium to strongly induce glyoxylate cycle activity. Whereas WT cells exhibited robust reporter activation, N88S cells showed a ~40–45% reduction (Figure 6B). These findings demonstrate that N88S mutant cells exhibit a marked defect to properly activate the glyoxylate cycle and adapt to energy demands during diauxic shift and stationary phases and when shifted to non-fermentable carbon sources. In agreement with this idea, N88S seipin-expressing cells showed impaired growth on acetate medium as sole carbon source (Figure 7).

We next investigated whether acetate utilization in these cells depends on the Krebs cycle, since both TCA and glyoxylate cycles are essential metabolic pathways for growth on acetate as the sole carbon source [46]. Moreover, proteomic and KEGG analyses further revealed reduced levels of proteins from the TCA cycle (Cit1p, Mdh2p), the glyoxylate cycle (Icl1p, Mls1p, Cit3p), and related processes (Mdh1p and Idp2p) (Figure 1). To test this directly, we compared the growth of WT and N88S mutant strains on glucose or acetate medium, in the presence or absence of sodium arsenite (NaAs, 250 μM). Sodium arsenite inhibits the TCA cycle by targeting the α-ketoglutarate dehydrogenase complex [46]. Arsenite did not exacerbate the growth defect of N88S seipin-expressing cells on acetate. Overall, these results demonstrate that acetate utilization in N88S mutant cells is not limited by the TCA cycle but rather by defective induction of the glyoxylate cycle, confirming its essential role in metabolic reprogramming and adaptation to non-fermentable carbon sources (Figure 7).

### 3.5. MPC1 Overexpression Exacerbates ER Stress but Does Not Contribute to ROS Generation upon N88S Seipin Mutation

**Figure 8:**
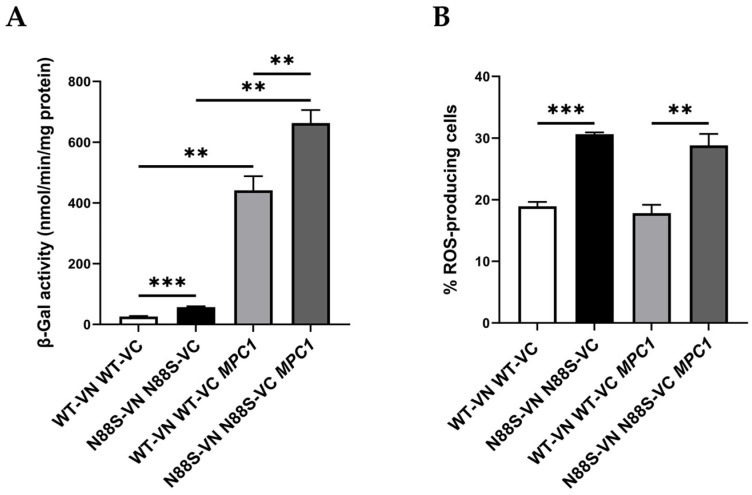
*MPC1* overexpression did not alter the levels of ROS production in WT and N88S seipin mutant cells. (**A**) Cells with the specified genotypes carrying pRS315-UPRE-LacZ or pRS315-UPRE-LacZ-ADH1pr-MPC1- ADH1t were grown in SC-glucose and allowed to reach the post-diauxic shift (PDS) phase. Protein extracts were prepared, and specific β-galactosidase (β-Gal) activity was measured using o-nitrophenyl-β-D-galactopyranoside (ONPG) as a substrate, with the amount of o-nitrophenol released indicating enzyme activity. At least 3 independent experiments for each strain were performed. (**B**) Cells with the indicated genotypes were grown in SC-glucose medium to stationary phase, and ROS levels were assessed in cells labeled with dihydroethidium (DHE) via flow cytometry using the FL3 channel. Cells were centrifuged and washed twice with PBS buffer. At least 3 independent experiments were conducted. ** *p* ≤ 0.01; *** *p* ≤ 0.001 (unpaired *t*-test with Welch’s correction).

Our results suggest that N88S mutant cells exhibit disrupted metabolic coupling between peroxisomal β-oxidation and mitochondrial anaplerosis, likely due to impaired activation of the glyoxylate cycle. While mammals lack the glyoxylate cycle, acetyl-CoA partitioning between peroxisomes, mitochondria, and cytosol is conserved, and defective anaplerotic support of mitochondria is a known feature of neurodegeneration. From our previous proteomics analysis [15], we also observed reduced protein levels of key metabolite transport/transfer proteins, including the mitochondrial inner membrane carnitine transporter Crc1p, which mediates carnitine-dependent transfer of acetyl-CoA from peroxisomes to mitochondria during FA β-oxidation, the mitochondrial succinate-fumarate transporter Sfc1p and the carnitine acetyltransferase Cat2p (Figure 1). Together, these findings suggest that N88S seipin-expressing cells may have reduced flux and utilization of peroxisome-derived acetyl-CoA in the glyoxylate cycle, along with defective transport into mitochondria. Consequently, acetyl-CoA or acetyl–carnitine may accumulate in the cytosol while acetyl-CoA becomes limiting in the mitochondria, thus restricting TCA cycle activity and energy production, ultimately leading to metabolic stress. To test whether this represents a metabolic bottleneck in acetyl-CoA supply, we overexpressed the mitochondrial pyruvate carrier (*MPC1*), a nuclear-encoded transporter that facilitates pyruvate uptake into mitochondria, thereby enhancing acetyl-CoA generation via the pyruvate dehydrogenase complex and boosting TCA cycle activity [47]. We first monitored ER stress activation using the UPRE-LacZ reporter. As shown in Figure 8A, *MPC1* overexpression triggered a strong UPR, with a ~13-fold and ~18-fold increase in β-galactosidase reporter activity in WT and N88S mutant cells, respectively. This indicates that while *MPC1* increases mitochondrial acetyl-CoA availability, it also triggers ER stress. Several mechanisms could explain how *MPC1* overexpression induces the UPR. First, excessive mitochondrial import of pyruvate may drive hyperactivation of the TCA cycle, leading to an imbalance in mitochondrial ATP and NADH production. Such shifts in mitochondrial redox state can disrupt ER homeostasis through the ER-mitochondria membrane contact sites, where calcium and lipid exchange are tightly regulated. Second, increased mitochondrial metabolism may elevate the demand for protein import, folding, and turnover in the organelle, indirectly contributing to ER stress. Third, metabolic rewiring through Mpc1p may alter lipid synthesis, since acetyl-CoA availability directly feeds into phospholipid and sterol biosynthetic pathways. In fact, perturbation in inositol metabolism and phospholipid homeostasis were previously established as ER stressors in N88S seipin-expressing cells [15]. Next, we assessed ROS generation using the DHE probe (Figure 8B). The results revealed that ROS levels remained unaltered in both WT and N88S cells upon *MPC1* overexpression. This suggests that enhanced incorporation of pyruvate and conversion to acetyl-CoA is not paramount to promote oxidative stress in the yeast model of N88S seipinopathy.

### 3.6. Cytosolic Misrouting of Acetyl-CoA Potentiates ER Stress and Contributes to Oxidative Damage in N88S Mutant Cells

**Figure 9:**
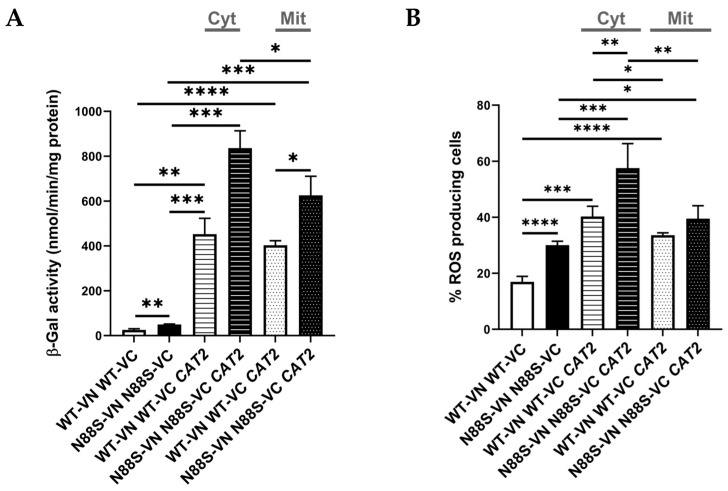
Changes in acetyl-CoA subcellular distribution triggers the UPR and ROS generation in WT and N88S seipin mutant cells. (**A**) Cells with the specified genotypes expressing pRS315-UPRE-LacZ, pRS315-UPRE-LacZ-ADH1pr-CAT2_cyt_-ADH1t or pRS315-UPRE-LacZ-ADH1pr-CAT2_mit_-ADH1t were grown in SC-glucose and allowed to reach the post-diauxic shift (PDS) phase. Protein extracts were prepared, and specific β-galactosidase (β-Gal) activity was measured using o-nitrophenyl-β-D-galactopyranoside (ONPG) as a substrate, with the amount of o-nitrophenol released indicating enzyme activity. At least 3 independent experiments for each strain were performed. (**B**) Cells were grown in SC-glucose medium to stationary phase, and ROS levels were assessed in cells labeled with dihydroethidium (DHE) via flow cytometry using the FL3 channel. Cells were centrifuged and washed twice with PBS buffer. At least 3 independent experiments were conducted. * *p* ≤ 0.05; ** *p* ≤ 0.01; *** *p* ≤ 0.001; **** *p* ≤ 0.0001 (unpaired *t*-test with Welch’s correction).

In *S. cerevisiae*, acetyl-CoA is synthesized in four subcellular compartments: the cytosol, mitochondria, peroxisomes, and nucleus. Among these, peroxisomes constitute a particularly rich source of acetyl-CoA owing to their exclusive role in FA β-oxidation [23,48]. Peroxisomal acetyl-CoA can either be metabolized within the glyoxylate cycle or transferred to mitochondria through the carnitine shuttle system. Because the peroxisomal membrane is impermeable to acetyl-CoA, this transfer requires conversion of acetyl-CoA to acetyl–carnitine by Cat2p, whose protein levels are decreased in N88S mutant cells (Figure 1). Once exported, acetyl–carnitine is reconverted to acetyl-CoA inside mitochondria, where it supports TCA cycle activity [49]. Based on our earlier observation that N88S mutant cells exhibit impaired utilization of peroxisome-derived acetyl-CoA, we hypothesized that dysfunction of the carnitine shuttle system may contribute to acetyl-CoA mislocalization and subsequent metabolic stress. To address this, we engineered two genetically modified backgrounds: one overexpressing the endogenous *CAT2* gene (hereafter CAT2_mit_) to enhance mitochondrial and peroxisomal carnitine acetyltransferase activity, and a second strain expressing a modified *CAT2* variant (hereafter CAT2_cyt_) engineered for exclusive cytosolic localization by removal of its native mitochondrial and peroxisomal targeting sequences [50]. This design allowed us to directly test whether manipulation of acetyl-CoA subcellular distribution impacts key disease phenotypes, including the ER stress response. Quantitative analysis of the UPRE-LacZ reporter activity (Figure 9A) revealed significant activation of the UPR in WT and N88S mutant cells expressing both *CAT2* variants. In the CAT2_mit_ background, the UPR reporter activity increased ~20-fold in WT and ~12-fold in N88S cells relative to their counterparts. Remarkably, the CAT2_cyt_ variant induced even stronger responses, with ~22-fold and ~17-fold activation in WT and N88S seipin-expressing cells, respectively (Figure 9A). These findings demonstrate that perturbations in acetyl-CoA compartmentalization, and particularly its cytosolic misrouting, is a more potent contributor to the activation of the ER stress response. This is consistent with a model in which excess cytosolic acetyl-CoA significantly perturbs ER homeostasis, likely through altered lipid metabolism or changes in redox balance. In parallel, we assessed ROS levels in these strains. The results showed that the expression of both *CAT2* variants increased the generation of ROS in WT and N88S seipin-expressing cells. Notably, in the N88S background, the expression of CAT2_cyt_ increased ROS levels (~90%) to a greater extent than those observed in N88S CAT2_mit_ -expressing cells (~33%) (Figure 9B).

**Figure 10:**
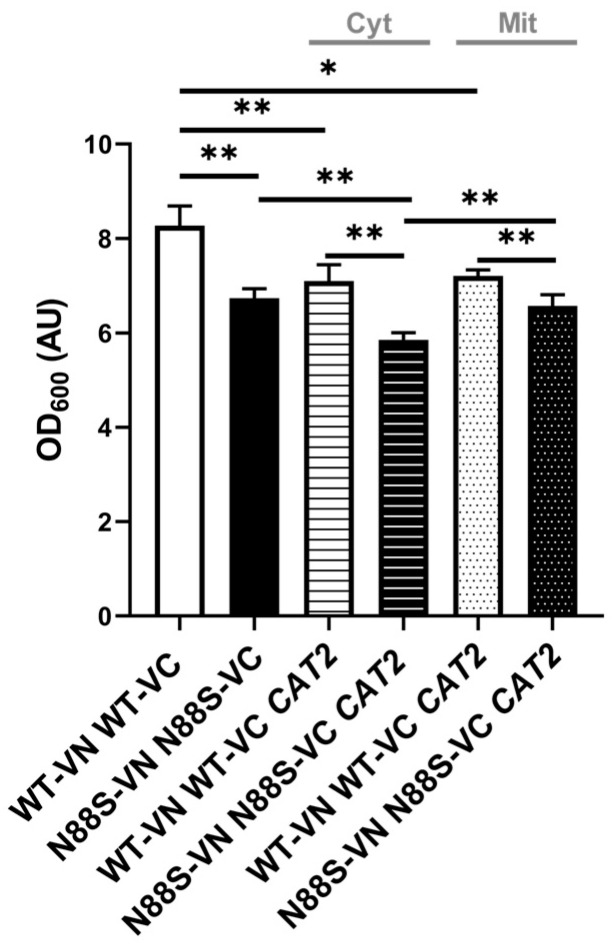
Mutant N88S cells expressing the cytosolic CAT2 showed growth defects at STAT phase. Overnight precultures of SC-glucose grown cells with the specified genotypes expressing pRS315-UPRE-LacZ, pRS315-UPRE-LacZ-ADH1pr-CAT2_cyt_-ADH1t or pRS315-UPRE-LacZ-ADH1pr-CAT2_mit_-ADH1t were diluted to OD_600_ = 0.15 in fresh medium. Cell density (OD_600_) was determined accordingly after a 48 h incubation period. Data were obtained from at least 3 independent experiments for each strain. * *p* ≤ 0.05; ** *p* ≤ 0.01 (unpaired *t*-test with Welch’s correction).

These results indicate that cytosolic accumulation of acetyl-CoA is not only a trigger for ER stress, but also a key driver of oxidative stress and damage, further supporting our initial hypothesis of impaired acetyl-CoA compartmentalization in disease-related metabolic dysfunctions. Notably, we observed a growth defect in N88S mutant cells overexpressing the cytosolic *CAT2* variant (CAT2_cyt_) once they reached stationary phase, but not for cells expressing the peroxisomal/mitochondrial variant (CAT2_mit_) (Figure 10).

**Figure 11:**
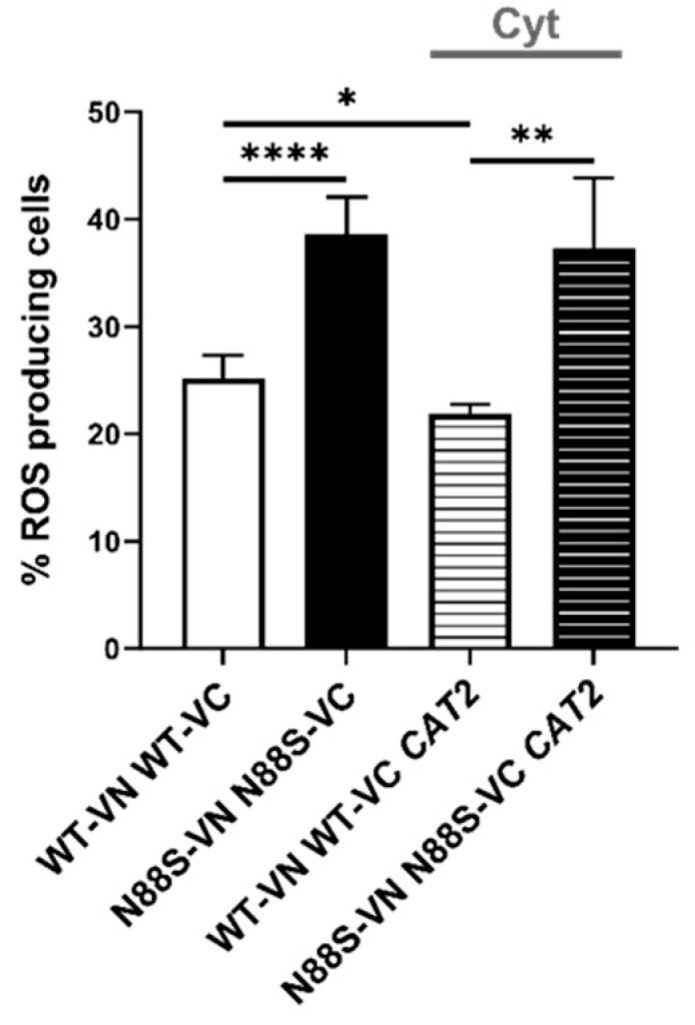
Carnitine supplementation does not affect ROS production in N88S seipin expressing cells. Cells with the indicated genotypes expressing pRS315-UPRE-LacZ or pRS315-UPRE-LacZ-ADH1pr-CAT2_cyt_-ADH1t were grown in SC-glucose medium supplemented with L-carnitine (100 mg/L) until stationary (STAT) phase, and ROS levels were assessed in cells labeled with dihydroethidium (DHE) via flow cytometry using the FL3 channel. Cells were centrifuged, washed twice with water, and diluted to OD_600_ = 0.5, at least 3 independent experiments for each strain. * *p* ≤ 0.05; ** *p* ≤ 0.01; **** *p* ≤ 0.0001 (unpaired *t*-test with Welch’s correction).

As *S. cerevisiae* is auxotrophic for carnitine, acetyl–carnitine shuttle activity is intrinsically limited under standard growth conditions. To determine whether exogenous carnitine supplementation could alleviate the stress phenotypes associated with cytosolic mislocalization of acetyl-CoA, CAT2_cyt_-overexpressing cells were cultured in media supplemented with L-carnitine. Supplementation failed to reduce ROS accumulation, with levels remaining at ~20–25% in WT and ~40% in N88S seipin-expressing cells upon overexpression of CAT2_cyt_ (Figure 11). These findings indicate that although carnitine supplementation modulates acetyl-CoA trafficking in yeast, it does not mitigate the cytotoxic consequences of its cytosolic accumulation. Rather, persistent ROS generation in N88S mutant cells arises from aberrant subcellular distribution and compartmentalization defects, likely reflecting impaired flux through the glyoxylate cycle.

### 3.7. Cytosolic Acetyl-CoA Fuels Lipid Biosynthesis and Promotes Oxidative Stress in N88S Seipin-Expressing Cells

**Figure 12:**
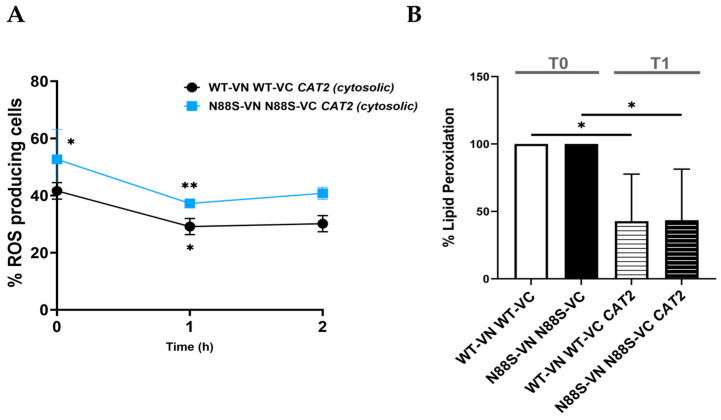
Cerulenin attenuates oxidative stress in WT and N88S seipin mutant cells expressing cytosolic *CAT2*. WT and N88S seipin mutant cells were grown in SC-glucose medium to stationary phase and incubated with 1 µg/mL cerulenin for 2 h (**A**) or 1 h (**B**) after which ROS levels were quantified using the DHE probe (**A**) and lipid peroxidation lipid peroxidation levels were monitored using the TBARS (thiobarbituric acid reactive substance) assay (**B**). Data represents the mean ± SEM from at least three independent experiments. * *p* ≤ 0.05; ** *p* ≤ 0.01 (two-way ANOVA for (**A**) and one-way ANOVA for (**B**)).

To investigate the downstream metabolic fate of mislocalized cytosolic acetyl-CoA, we next examined its potential diversion into lipid biosynthesis pathways. Because acetyl-CoA is the primary substrate for FA synthesis, we employed the well-characterized fatty acid synthase (FAS) inhibitor cerulenin, a covalent and irreversible inhibitor of the β-ketoacyl-ACP synthase domain, which effectively blocks the elongation of acyl chains and suppresses de novo lipogenesis [51,52]. Treatment of WT and N88S *CAT2*_cyt_-expressing strains with cerulenin (1 µg/mL) reduced ROS levels after 1 h (Figure 12A). Importantly, this was associated with decreased lipid peroxidation (Figure 12B).

**Figure 13:**
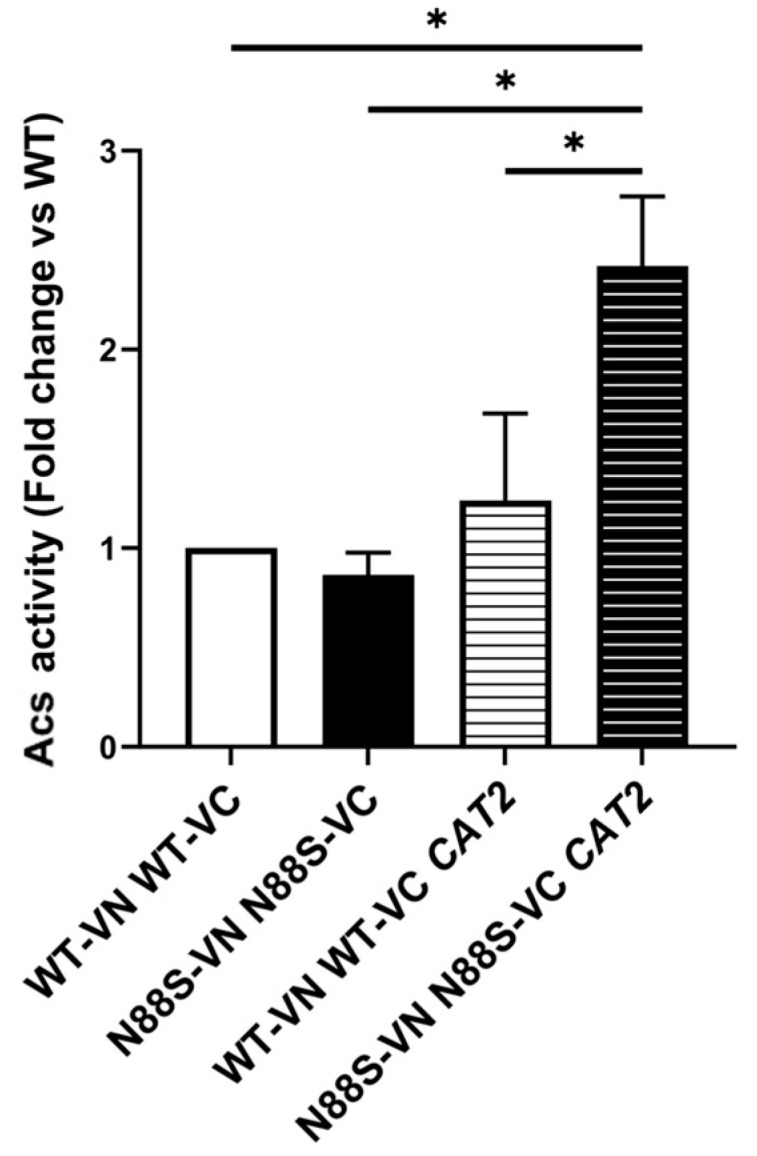
The activity of acetyl–coenzyme A synthetase (Acs) was increased in N88S mutant cells overexpressing cytosolic *CAT2*. Acs enzymatic activity was measured in cells grown in SC-glucose medium to PDS phase. Results are mean ± SD of at least three independent experiments. * *p* ≤ 0.05 (unpaired *t*-test with Welch’s correction).

These results provide strong evidence that excess cytosolic acetyl-CoA is actively funneled into FA biosynthesis and that this metabolic flux contributes to oxidative stress through enhanced lipid peroxidation. Finally, since acetyl-CoA can serve as a substrate for epigenetic regulation via histone acetylation [53], we assessed global histone H3 acetylation by Western blot. No significant differences were observed between WT and N88S seipin-expressing cells upon overexpression of cytosolic *CAT2* (data not shown), indicating that histone acetylation does not act as a major sink for excess cytosolic acetyl-CoA under these conditions. Consistent with this, acetyl–coenzyme A synthetase (Acs) activity, which supplies acetyl-CoA for histone acetylation, was increased in N88S mutant cells overexpressing cytosolic *CAT2* relative to its N88S mutant counterpart, suggesting that acetyl-CoA availability for histone acetylation is not limiting (Figure 13). On the other hand, excessive activation of the acetyl–coenzyme A synthetase at PDS phase likely feeds further the cytosolic acetyl-CoA pool and may contribute to its deleterious effects in N88S seipin-expressing cells. Overall, we speculate that impaired acetyl-CoA compartmentalization may exacerbate lipid imbalance and directly contribute to UPR activation and ER stress observed in this model and mammalian systems [10], independently of seipin misfolding per se, by altering phospholipid synthesis, membrane composition, and lipid flux at the ER. It is also possible that lipid imbalance is reflected in the LD heterogeneity observed in both yeast [54] and mammalian system [55]. In this framework, the activation of autophagy observed in mammalian models [55] may arise as a metabolic stress response to persistent defects in lipid homeostasis, redox balance, and organelle function, rather than solely as a mechanism to clear seipin aggregates, potentially linking metabolic uncoupling to broader cellular dysfunction relevant to motor neurodegeneration.

Altogether, our results indicate that metabolic dysfunction exhibited by N88S mutant cells are not due to a global deficit in acetyl-CoA synthesis, but rather to its impaired compartmentalization and misrouting. Specifically, peroxisome-derived acetyl-CoA fails to reach mitochondria efficiently and instead accumulates in the cytosol, where it is redirected into lipid biosynthesis, fueling ROS production and ER stress. Therefore, we posit that cytosolic acetyl-CoA-driven lipogenesis is a key contributor to cellular dysfunction in N88S mutant cells.

## 4. Conclusions

**Figure 14:**
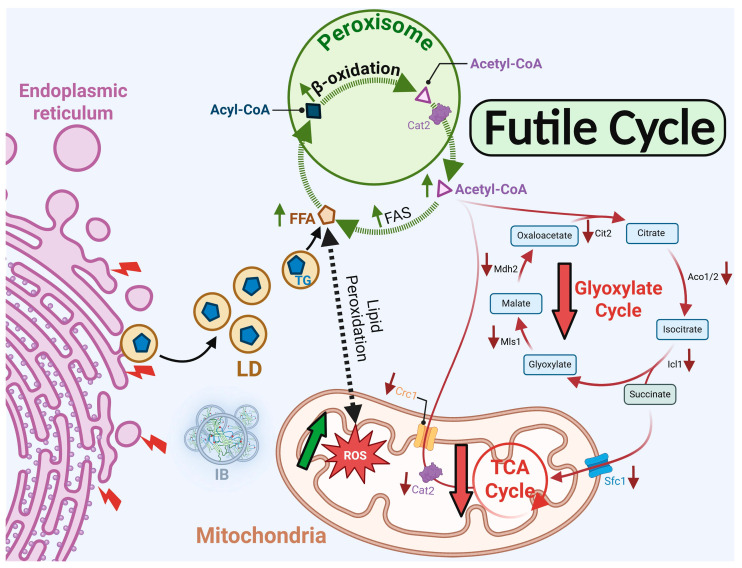
Metabolic dysfunctions imparted by the N88S seipin mutation. Schematic summary of the interconnected metabolic and cellular defects caused by N88S seipin. The mutation impairs phospholipid and inositol metabolism. Concurrently, cytosolic acetyl-CoA becomes miscompartmentalized due to inefficient mitochondrial import via the carnitine-dependent shuttle, reducing flux into the TCA and glyoxylate cycles. Excess cytosolic acetyl-CoA is redirected into de novo fatty acid synthesis, followed by peroxisomal β-oxidation, establishing a highlighted futile FA synthesis-oxidation cycle that amplifies lipid peroxidation and ROS production. Together, impaired lipid and inositol metabolism, oxidative stress, and misfolded seipin accumulation overwhelm ERAD and UPR pathways, generating a self-reinforcing cycle of ER stress and oxidative damage.

Our study demonstrates that the N88S seipin mutation disrupts the interplay between lipid and protein metabolism, linking altered phospholipid homeostasis, miscompartmentalized acetyl-CoA, and oxidative stress to the cellular pathophysiology of seipinopathy. Using our yeast model of N88S seipinopathy, we previously showed that inositol and neutral lipid metabolism, as well as cellular iron homeostasis, are significantly impaired [15]. Central to this phenotype is the accumulation of phosphatidic acid (PA), reflecting a reduction in phospholipid biosynthetic flux and impaired inositol metabolism. This metabolic imbalance interferes with the activity of the Opi1p transcriptional repressor, resulting in derepression of *INO1* and elevated Ino1p levels [15]. Notably, PA accumulation and altered inositol metabolism contribute to ER stress independently of seipin misfolding, highlighting that lipid dysregulation alone can act as a driver of ER dysfunction [15]. In addition to perturbations in phospholipid and inositol metabolism, this study identified a previously unrecognized metabolic feedback loop that amplifies oxidative stress through mislocalized cytosolic acetyl-CoA. In N88S mutant cells, acetyl-CoA is inefficiently imported into mitochondria via the carnitine-dependent shuttle and is poorly channeled into the glyoxylate and TCA cycles. Instead, it is redirected into de novo FA lipogenesis, and these FAs are subsequently processed in peroxisomes via β-oxidation, regenerating cytosolic acetyl-CoA and establishing a futile cycle (Figure 14). This cycle exacerbates lipid peroxidation and ROS production. In fact, short-term inhibition of FA synthase partially mitigates oxidative stress, confirming that redirected lipogenesis directly contributes to cellular damage. This mechanistic framework explains the elevated ROS levels observed in N88S cells and links cytosolic acetyl-CoA miscompartmentalization to excessive FA turnover and peroxisome proliferation. The N88S mutation therefore produces a dual pathogenic signature. As a proteinopathy, it is characterized by misfolded seipin accumulation in the ER, which overwhelms the ERAD system and the UPR. As a lipidopathy, it arises from both disruptions in inositol and phospholipid homeostasis, which are key drivers of the ER stress response [15], and from impaired acetyl-CoA flux that fuels futile cycles of FA synthesis and oxidation, thereby amplifying oxidative stress and cellular damage. Together, these intertwined defects establish a self-reinforcing network in which ER stress, lipid peroxidation, and ROS generation likely potentiate one another, providing a mechanistic link between metabolic dysfunction and lipid storage phenotypes in N88S seipinopathy that is a common feature shared with other neurodegenerative disorders. These findings also redefine a framework for potential therapeutic strategies for these motor neuron diseases. Interventions should aim not only to mitigate ROS or inhibit lipogenesis but also to restore proper metabolic compartmentalization and interrupt the self-amplifying lipid futile cycle. Enhancing mitochondrial import and utilization of acetyl-CoA via the Cat2p-assisted carnitine shuttle can relieve cytosolic acetyl-CoA accumulation and reduce substrate flow into de novo FA synthesis. We cannot discard the possibility that Yat1p and Yat2p carnitine acetyltransferases may also be playing a role in this process. Redirecting cytosolic acetyl-CoA into sterol or phospholipid biosynthesis can buffer its accumulation while restoring membrane lipid homeostasis. Stimulating phospholipid synthesis, which is impaired in N88S cells [15], may serve a dual purpose as it may sequester excess acetyl-CoA and stabilize ER architecture, potentially alleviating ER stress associated with seipin misfolding. Additionally, modulating lipogenic transcriptional programs through AMPK activation or SREBP suppression in mammalian cells may further recalibrate lipid synthesis to match the cellular energy and redox demands.

Importantly, peroxisome proliferation in N88S mutant cells appears to be a compensatory adaptation rather than a simple consequence of lipotoxicity. Peroxisomal proliferation increases the capacity to promote the β-oxidation of FA derived from mislocalized acetyl-CoA, partially alleviating lipid accumulation. Peroxisomes also contribute to redox homeostasis through regulation of glutathione metabolism, which can compensate, at least partially, for diminished catalase activity and elevated ROS levels [16]. Therapeutic approaches that fine-tune peroxisomal function while redirecting acetyl-CoA flux could therefore synergistically reduce oxidative damage and restore metabolic balance. Taken together, a multifaceted strategy that promotes mitochondrial utilization of acetyl-CoA, enhances phospholipid synthesis, fine-tunes lipogenic transcription, and optimizes peroxisomal function provides a rational framework to disrupt the futile cycle at multiple nodes. Such an approach not only mitigates lipotoxicity and oxidative stress but also restores lipid metabolism, preserves membrane integrity, and supports adaptive cellular mechanisms critical for the survival of N88S seipin-expressing cells under physiological and environmental stress.

## 5. Limitations of the Model

While the glyoxylate cycle itself is yeast-specific, the central focus of our work is not the pathway per se, but rather the regulation of acetyl-CoA compartmentalization and inter-organelle metabolic coupling, which are evolutionarily conserved processes. In both yeast and mammalian cells, peroxisomes generate acetyl-CoA through fatty acid β-oxidation, and its proper distribution between peroxisomes, mitochondria, and cytosol is essential for maintaining redox balance and lipid homeostasis. In mammalian neurons, although acetyl-CoA cannot enter a glyoxylate shunt, it must still be efficiently transferred or metabolically integrated to avoid cytosolic accumulation and aberrant lipid synthesis. Dysregulated peroxisome-mitochondria communication and altered lipid metabolism are well-established contributors to neurodegenerative disorders. Here, the glyoxylate cycle serves as a metabolic readout of impaired acetyl-CoA routing, rather than as a direct disease-equivalent pathway. The key conserved principle emerging from our study is that N88S seipin expression disrupts the balance between oxidative utilization and cytosolic lipogenic diversion of acetyl-CoA. This misallocation promotes lipid-driven oxidative stress and ER dysfunction, which are key mechanisms that are well known drivers of neurodegeneration, and highly relevant to human motor neuron pathology. Moreover, peroxisomal dysfunction is increasingly recognized as a contributing factor in neurological disease, including disorders characterized by lipid imbalance and oxidative stress. Although peroxisomal metabolic outputs differ between yeast and neurons, the requirement for coordinated organelle cross-talk to maintain redox and lipid homeostasis is still conserved. Therefore, our yeast model still provides a genetically tractable system to dissect how N88S mutant seipin perturbs this metabolic coupling at a mechanistic level.

In summary, our findings provide an integrated mechanistic model for N88S seipinopathy, demonstrating how subtle disruptions in phospholipid metabolism, acetyl–CoA miscompartmentalization, and oxidative stress converge to perturb both protein and lipid homeostasis. This work underscores the central role of seipin in coordinating lipid balance, redox homeostasis, and ER function, and highlights the importance of targeting metabolic compartmentalization and peroxisomal adaptation as potential therapeutic avenues to tackle the cellular dysfunctions in N88S seipin-associated motor neuron diseases.

## Data Availability

All related data is presented in this study. Relevant materials or supplies will be made available under a material transfer agreement. No code was used in this study.

## Supplementary Materials

The following supporting information can be downloaded at: https://www.mdpi.com/article/10.3390/cells15050395/s1, Figure S1. The loss of peroxisomes leads to an increase in total glutathione levels in N88S seipin-expressing cells. Figure S2. The raw data of Western blot assays. The figures display all blots used in this study and in dashed red the selected areas shown in the main text; Table S1. Yeast strains used in this study. Table S2. Plasmids used in this study. Table S3. Conditions for the measurement of glyoxylate and TCA cycle enzyme activities [16,46,56].

## Abbreviations

The following abbreviations are used in this manuscript:

Acs	Acetyl Coenzyme A Synthetase
ADH1pr	ADH1 Promoter
ADH1t	ADH1 Terminator
ANOVA	Analysis Of Variance
Ats	Amino-Terminal Signal (Context: Targeting Sequences)
ATP	Adenosine Triphosphate
**β**-Gal	Beta-Galactosidase
BIFC	Bimolecular Fluorescence Complementation
BSA	Bovine Serum Albumin
CAT2_cy_	Cytosolic Variant of Carnitine Acetyltransferase Cat2p
CAT2_mit_	Mitochondrial/Peroxisomal Targeting Variant of Cat2p
Cit2p	Citrate Synthase 2
Deps	Differentially Expressed Proteins
DHE	Dihydroethidium
DIC	Differential Interference Contrast
DNA	Deoxyribonucleic Acid
DTNB	Ellman’s Reagent (5,5′-Dithiobis-2-Nitrobenzoic Acid)
EDTA	Ethylenediaminetetraacetic Acid
ER	Endoplasmic Reticulum
ERAD	ER-Associated Degradation
EXP	Exponential Phase
FA	Fatty Acid
FAS	Fatty Acid Synthase
Fe-S	Iron–Sulfur
FL3	Flow Cytometry Fluorescence Channel 3
GSH	Reduced Glutathione
GSSG	Oxidized Glutathione
GO	Gene Ontology
H3	Histone H3
IB	Inclusion Body
Icl1p	Isocitrate Lyase
INO1	Inositol-1-Phosphate Synthase Gene
KEGG	Kyoto Encyclopedia Of Genes And Genomes
KO	Knockout
LD	Lipid Droplet
MDA	Malondialdehyde
Mls1p	Malate Synthase
MPC1	Mitochondrial Pyruvate Carrier 1
MTS	Mitochondrial Targeting Signal
N88S	Pathogenic Asparagine-To-Serine Mutation In Seipin
ONPG	O-Nitrophenyl-Β-D-Galactopyranoside
ORF	Open Reading Frame
PA	Phosphatidic Acid
PBS	Phosphate-Buffered Saline
PCR	Polymerase Chain Reaction
PDS	Post-Diauxic Shift
PEX3	Peroxin 3
PEX19	Peroxin 19
Pot1p	Ketoacyl-Coa Thiolase
Pox1p	Acyl-Coa Oxidase
PTS1	Peroxisomal Targeting Signal 1
ROS	Reactive Oxygen Species
SC	Synthetic Complete
SD	Standard Deviation
SE	Sterol Ester
TG	Triacylglycerol
TCA	Tricarboxylic Acid (Cycle)
UPR	Unfolded Protein Response
UPRE-Lacz	Unfolded Protein Response Element–Lacz Transcriptional Reporter
VC	C-Terminal Fragment Of Venus Fluorescent Protein
VN	N-Terminal Fragment Of Venus Fluorescent Protein
WT	Wild-Type
YPD	Yeast Extract Peptone Dextrose Medium
YNB	Yeast Nitrogen Base
YFP	Yellow Fluorescent Protein
YEASTRACT	Yeast Transcriptional Regulatory Network Analysis Tool
