# Supplementary material for: Impaired Acetyl-CoA Compartmentalization Drives a Futile Lipogenic–Oxidative Cycle in N88S Seipinopathy
Source: Cells. 2026 Feb 24;15(5):395. doi: 10.3390/cells15050395 (PMC12984136; doi:10.3390/cells15050395)
Supplement: Supplementary file 1 [file cells-15-00395-s001.zip › cells-4153046-supplementary/Figure S1.pdf]

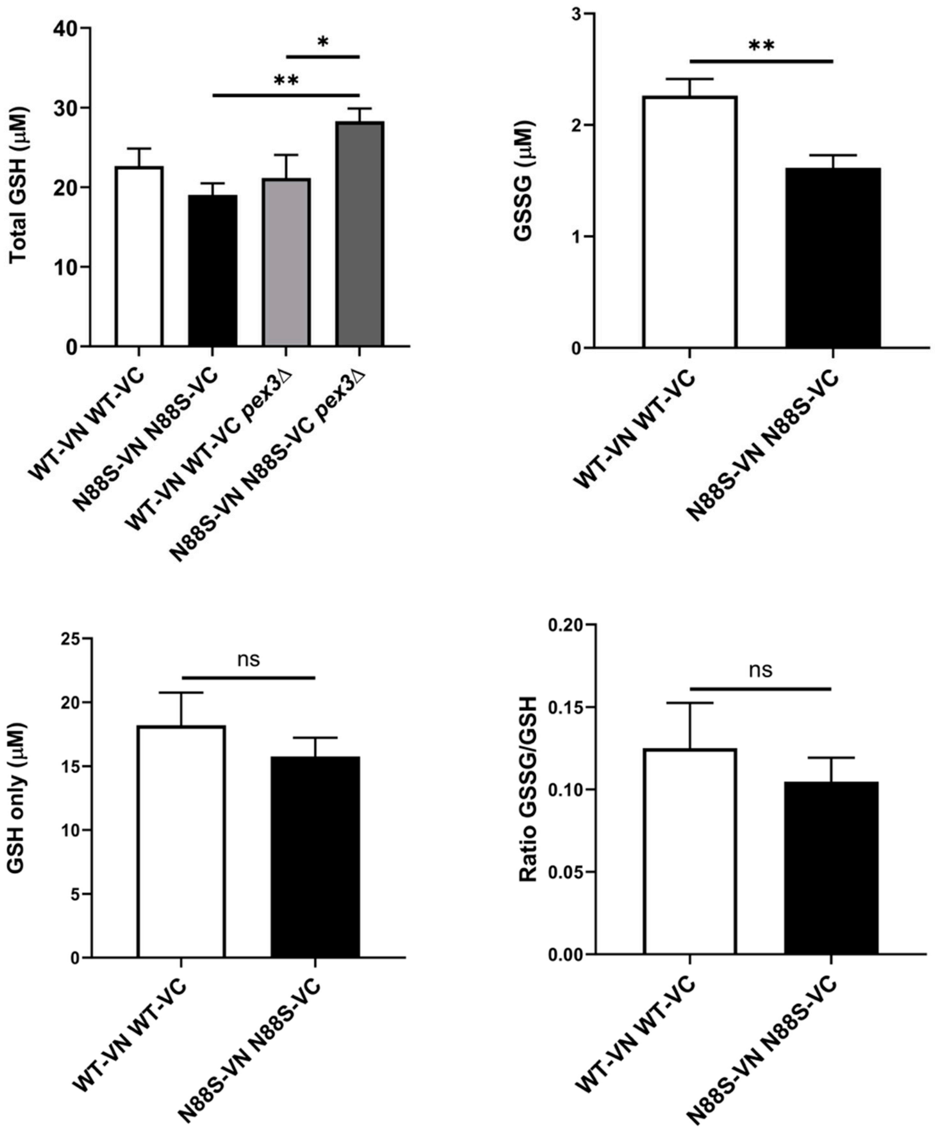

Figure S1. The loss of peroxisomes leads to an increase in total glutathione levels in N88S seipin-expressing cells. Total glutathione, oxidized (GSSG), reduced (GSH) glutathione levels and the ratio of GSSG to GSH were determined in cells grown in SC-glucose medium to PDS phase. At least 3 independent experiments were performed, as described in Materials and Methods. * $p \leq 0.05$; ** $p \leq 0.01$; ns - non-significant (unpaired *t*-test with Welch´s correction).
