# Supplementary material for: Impaired Acetyl-CoA Compartmentalization Drives a Futile Lipogenic–Oxidative Cycle in N88S Seipinopathy
Source: Cells. 2026 Feb 24;15(5):395. doi: 10.3390/cells15050395 (PMC12984136; doi:10.3390/cells15050395)
Supplement: Supplementary file 1 [file cells-15-00395-s001.zip › cells-4153046-supplementary/revised Tables Moreira et al_2025.pdf]

Table S1. Yeast strains used in this study.

Strain	Genotype	Source/Reference
**WT-VN WT-VC**	W303α *ldb16*Δ::natNT2 *fld1*Δ::hphmx4<br>pRS306-GPDpr-WTh*BSCL2*-VN-CYC1t<br>pRS413-GPDpr-WTh*BSCL2*-VC-CYC1t	(16)
**N88S-VN N88S-VC**	W303α *ldb16*Δ::natNT2 *fld1*Δ::hphmx4<br>pRS306-GPDpr-N88Sh*BSCL2*-VN-CYC1t<br>pRS413-GPDpr-N88Sh*BSCL2*-VC-CYC1t	(16)
**WT-VN WT-VC *pex3*Δ**	W303α *ldb16*Δ::natNT2 *fld1*Δ::hphmx4<br>pRS306-GPDpr-WTh*BSCL2*-VN-CYC1t<br>pRS413-GPDpr-WTh*BSCL2*-VC-CYC1t<br>*pex3*Δ::KANMX6	This study
**N88S-VN N88S-VC *pex3*Δ**	W303α *ldb16*Δ::natNT2 *fld1*Δ::hphmx4<br>pRS306-GPDpr-N88Sh*BSCL2*-VN-CYC1t<br>pRS413-GPDpr-N88Sh*BSCL2*-VC-CYC1t<br>*pex3*Δ::KANMX6	This study
**WT-VN WT-VC PTS1**	W303α *ldb16*Δ::natNT2 *fld1*Δ::hphmx4<br>pRS306-GPDpr-WTh*BSCL2*-VN-CYC1t<br>pRS413-GPDpr-WTh*BSCL2*-VC-CYC1t<br>pRS315-ADH1p-mCherry-PTS1	This study
**N88S-VN N88S-VC PTS1**	W303α *ldb16*Δ::natNT2 *fld1*Δ::hphmx4<br>pRS306-GPDpr-N88Sh*BSCL2*-VN-CYC1t<br>pRS413-GPDpr-N88Sh*BSCL2*-VC-CYC1t<br>pRS315-ADH1p-mCherry-PTS1	This study
**N88S-VN N88S-VC *pex3*Δ PTS1**	W303α *ldb16*Δ::natNT2 *fld1*Δ::hphmx4<br>pRS306-GPDpr-N88Sh*BSCL2*-VN-CYC1t<br>pRS413-GPDpr-N88Sh*BSCL2*-VC-CYC1t<br>*pex3*Δ::KANMX6<br>pRS315-ADH1p-mCherry-PTS1	This study
**N88S-VN N88S-VC *pex19*Δ PTS1**	W303α *ldb16*Δ::natNT2 *fld1*Δ::hphmx4<br>pRS306-GPDpr-N88Sh*BSCL2*-VN-CYC1t<br>pRS413-GPDpr-N88Sh*BSCL2*-VC-CYC1t<br>*pex19*Δ::KANMX6<br>pRS315-ADH1p-mCherry-PTS1	This study
**WT-VN WT-VC UPRE-LacZ**	W303α *ldb16*Δ::natNT2 *fld1*Δ::hphmx4<br>pRS306-GPDpr-WTh*BSCL2*-VN-CYC1t<br>pRS413-GPDpr-WTh*BSCL2*-VC-CYC1t<br>pRS315-UPRE-LacZ	(16)
**N88S-VN N88S-VC UPRE-LacZ**	W303α *ldb16*Δ::natNT2 *fld1*Δ::hphmx4	(16)

	pRS306-GPDpr-N88Sh*BSCL2*-VN-CYC1t<br>pRS413-GPDpr-N88Sh*BSCL2*-VC-CYC1t<br>pRS315-UPRE-LacZ	
**WT-VN WT-VC *pex3*Δ UPRE-LacZ**	W303α *ldb16*Δ::natNT2 *fld1*Δ::hphmx4<br>pRS306-GPDpr-WTh*BSCL2*-VN-CYC1t<br>pRS413-GPDpr-WTh*BSCL2*-VC-CYC1t<br>*pex3*Δ::KANMX6<br>pRS315-UPRE-LacZ	This study
**N88S-VN N88S-VC *pex3*Δ UPRE-LacZ**	W303α *ldb16*Δ::natNT2 *fld1*Δ::hphmx4<br>pRS306-GPDpr-N88Sh*BSCL2*-VN-CYC1t<br>pRS413-GPDpr-N88Sh*BSCL2*-VC-CYC1t<br>*pex3*Δ::KANMX6<br>pRS315-UPRE-LacZ	This study
**WT-VN WT-VC *CIT2*-LacZ**	W303α *ldb16*Δ::natNT2 *fld1*Δ::hphmx4<br>pRS306-GPDpr-WTh*BSCL2*-VN-CYC1t<br>pRS413-GPDpr-WTh*BSCL2*-VC-CYC1t<br>*CIT2*-LacZ-KANMX4	This study
**N88S-VN N88S-VC *CIT2*-LacZ**	W303α *ldb16*Δ::natNT2 *fld1*Δ::hphmx4<br>pRS306-GPDpr-N88Sh*BSCL2*-VN-CYC1t<br>pRS413-GPDpr-N88Sh*BSCL2*-VC-CYC1t<br>*CIT2*-LacZ-KANMX4	This study
**WT-VN WT-VC UPRE-LacZ *MPC1***	W303α *ldb16*Δ::natNT2 *fld1*Δ::hphmx4<br>pRS306-GPDpr-WTh*BSCL2*-VN-CYC1t<br>pRS413-GPDpr-WTh*BSCL2*-VC-CYC1t<br>pRS315-UPRE-LacZ-ADH1pr-*MPC1*-ADH1t	This study
**N88S-VN N88S-VC UPRE-LacZ *MPC1***	W303α *ldb16*Δ::natNT2 *fld1*Δ::hphmx4<br>pRS306-GPDpr-N88Sh*BSCL2*-VN-CYC1t<br>pRS413-GPDpr-N88Sh*BSCL2*-VC-CYC1t<br>pRS315-UPRE-LacZ-ADH1pr-*MPC1*- ADH1t	This study
**WT-VN WT-VC UPRE-LacZ *CAT2*$_{cyt}$**	W303α *ldb16*Δ::natNT2 *fld1*Δ::hphmx4<br>pRS306-GPDpr-WTh*BSCL2*-VN-CYC1t<br>pRS413-GPDpr-WTh*BSCL2*-VC-CYC1t<br>pRS315-UPRE-LacZ-ADH1pr-*CAT2*$_{cyt}$- ADH1t	This study
**N88S-VN N88S-VC UPRE-LacZ *CAT2*$_{cyt}$**	W303α *ldb16*Δ::natNT2 *fld1*Δ::hphmx4<br>pRS306-GPDpr-N88Sh*BSCL2*-VN-CYC1t<br>pRS413-GPDpr-N88Sh*BSCL2*-VC-CYC1t<br>pRS315-UPRE-LacZ-ADH1pr-*CAT2*$_{cyt}$- ADH1t	This study
**WT-VN WT-VC UPRE-LacZ *CAT2*$_{mit}$**	W303α *ldb16*Δ::natNT2 *fld1*Δ::hphmx4<br>pRS306-GPDpr-WTh*BSCL2*-VN-CYC1t<br>pRS413-GPDpr-WTh*BSCL2*-VC-CYC1t<br>pRS315-UPRE-LacZ-ADH1pr-*CAT2*$_{mit}$ ADH1t	This study
**N88S-VN N88S-VC UPRE-LacZ *CAT2*$_{mit}$**	W303α *ldb16*Δ::natNT2 *fld1*Δ::hphmx4	This study

pRS306-GPDpr-N88Sh*BSCL2*-VN-CYC1t
pRS413-GPDpr-N88Sh*BSCL2*-VC-CYC1t
pRS315-UPRE-LacZ-ADH1pr-*CAT2*$_{mit}$- ADH1t

GPDpr, GPD promoter; **ADH1pr**, ADH1 promoter; **ADH1t**, ADH1 terminator; **CYC1t**, CYC1 terminator; **VN**, Venus N-terminal fragment; **VC**, Venus C-terminal fragment.

Table S2. Plasmids used in this study.

Plasmids	Backbone	Description	Source/ Reference
**VT6**	pRS413	pRS413-GPDpr-WTseipin-VC-CYC1t	(16)
**VT7**	pRS413	pRS413-GPDpr-N88Sseipin-VC-CYC1t	(16)
**pFA6a-KANMX6**	pFA6a	ORF deletion	(31)
**PTS1-mCherry**	pFL24	pFL24-ADH1p-mCherry-PTS1	(56)
***UPRE*-LacZ**	pRS315	pRS315-UPRE-LacZ	(16)
***MPC1* OEx**	pRS315	pRS315- UPRE-LacZ-ADH1pr-*MPC1*-ADH1t	This study
***CIT2*-LacZ**	-	*CIT2*-LacZ-KANMX4	Dr Zhengchang Liu, University of New Orleans
***CAT2*$_{mit}$**	pRS315	pRS315-UPRE-LacZ-ADH1pr-*CAT2*$_{mit}$-ADH1t	This study
***CAT2*$_{cyt}$**	pRS315	pRS315-UPRE-LacZ-ADH1pr-*CAT2*$_{cyt}$-ADHt	This study

Table S3. Conditions for the measurement of glyoxylate and TCA cycle enzyme activities.

Enzyme	Buffer (mM)	Other addition(s) [brand] (mM)	Wavelength (nm)	$\mathcal{E}$ (M^{-1} cm^{-1} × 10^{-8})
**Citrate synthase (EC 4.1.3.7)**	100 Tris–HCl, pH 8.5	Oxaloacetate [Sigma-Aldrich] (0.25), acetyl-CoA [Sigma-Aldrich] (0.04), DTNB [Sigma-Aldrich] (0.1)	412	13.60
**NAD-dependent isocitrate dehydrogenase (EC 1.1.1.41)**	100 Tris–HCl, pH 7.4	D,L-Isocitrate [Sigma-Aldrich] (5), NAD [Sigma-Aldrich] (1.7),AMP [Thermo Scientific] (0.8), MgCl2 [Merck] (3)	340	6.22
**NADP-dependent isocitrate dehydrogenase (EC 1.1.1.42)**	100 Tris–HCl, pH 7.4	D,L-Isocitrate (5), NADP [Sigma-Aldrich] (0.6), MgCl2 (3)	340	6.22
**Malate dehydrogenase (EC 1.1.1.37)**	100 Tris–HCl, pH 7.4	Oxaloacetate (0.15), NADH (0.1)	340	6.22
**Isocitrate lyase (EC 4.1.3.1)**	100 KPi, pH 7.4	D,L-Isocitrate (8), cysteine [Sigma-Aldrich] (2), phenylhydrazine [Sigma-Aldrich] (3.3), MgCl2 (3)	324	17.00
**Malate synthase (EC 4.1.3.2)**	100 Tris–HCl, pH 8.5	Glyoxylate (5), acetyl-CoA (0.1), MgCl2 (10), DTNB (0.33)	412	13.6
