# Supplementary material for: Impaired Acetyl-CoA Compartmentalization Drives a Futile Lipogenic–Oxidative Cycle in N88S Seipinopathy
Source: Cells. 2026 Feb 24;15(5):395. doi: 10.3390/cells15050395 (PMC12984136; doi:10.3390/cells15050395)
Supplement: Supplementary file 1 [file cells-15-00395-s001.zip › cells-4153046-supplementary/Supplementary Figure Cells_blots.pdf]

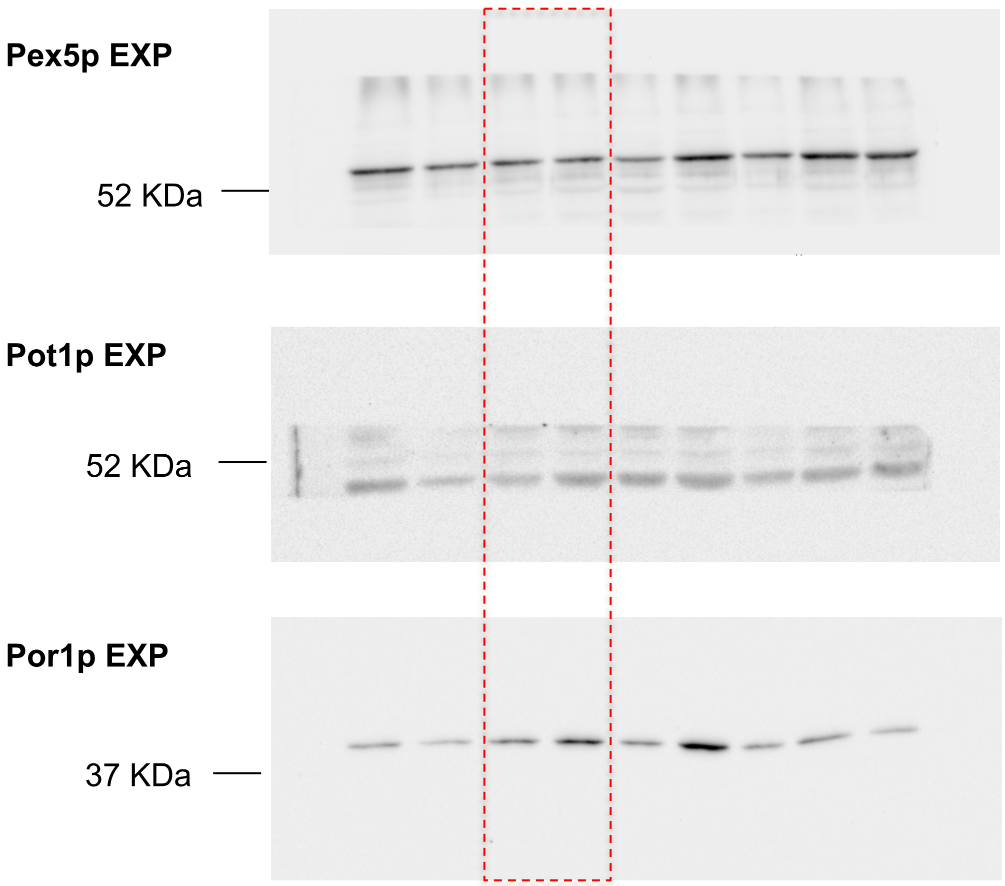
Pex5p EXP
52 KDa
Pot1p EXP
52 KDa
Por1p EXP
37 KDa

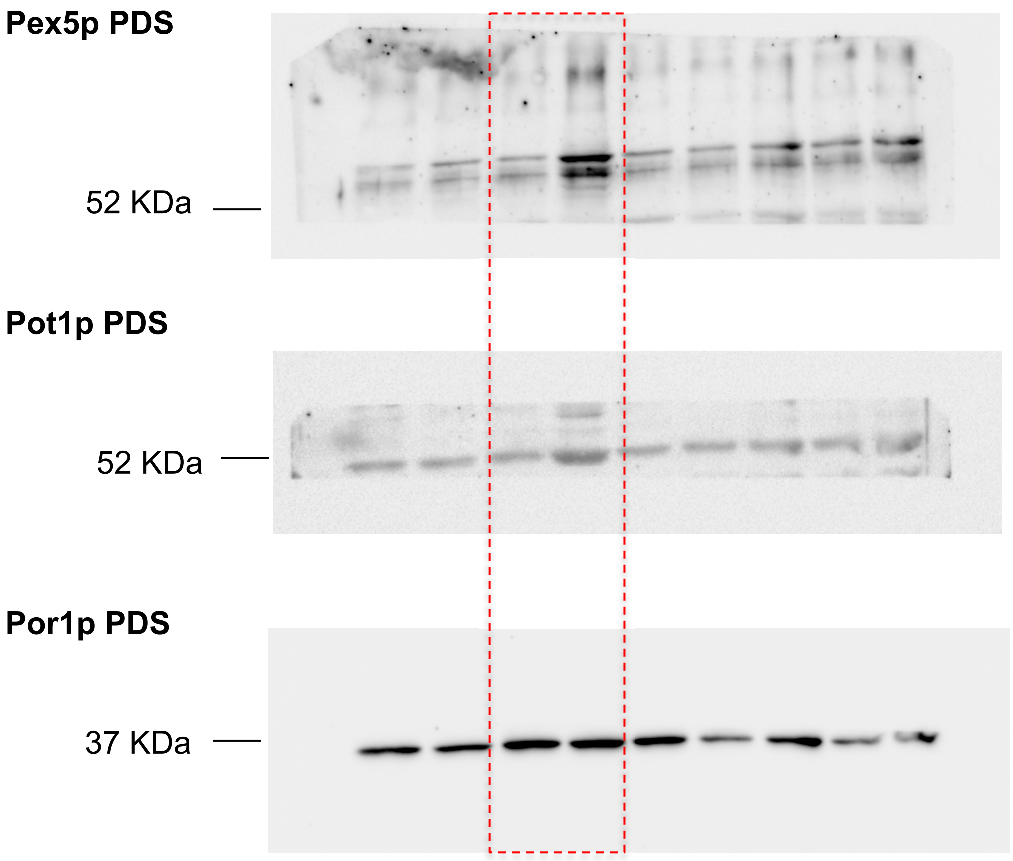

Supplementary Figure S2. The raw data of Western blot assays. The figures display all blots used in this study and in dashed red the selected areas shown in the main text.
